# The Impact of *Chlamydia trachomatis* on Male Infertility: A Systematic Review and Meta-Analysis

**DOI:** 10.1093/ofid/ofaf782

**Published:** 2025-12-23

**Authors:** Nicola Luigi Bragazzi, Valerie Bosch Castells, Qi Deng, Grégoire Ranson, Edward Thommes, Jianhong Wu, Sandra S Chaves

**Affiliations:** Laboratory for Industrial and Applied Mathematics (LIAM), Department of Mathematics and Statistics, York University, Toronto, Ontario, Canada; Department of Food and Drugs, University of Parma, Parma, Italy; Department of Clinical Pharmacy, Saarland University, Saarbrücken, Germany; Sanofi, Lyon, France; Laboratory for Industrial and Applied Mathematics (LIAM), Department of Mathematics and Statistics, York University, Toronto, Ontario, Canada; Laboratory for Industrial and Applied Mathematics (LIAM), Department of Mathematics and Statistics, York University, Toronto, Ontario, Canada; Institut Camille Jordan (Inria), Université Claude Bernard Lyon 1, Villeurbanne, France; Sanofi, Toronto, Canada; University of Guelph, Guelph, Ontario, Canada; Laboratory for Industrial and Applied Mathematics (LIAM), Department of Mathematics and Statistics, York University, Toronto, Ontario, Canada; Sanofi, Lyon, France

**Keywords:** *chlamydia trachomatis*, male infertility, meta-analysis, reproductive health, systematic review

## Abstract

**Background:**

*Chlamydia trachomatis* (CT) is a common sexually transmitted infection, yet its contribution to male infertility remains incompletely understood.

**Methods:**

A systematic review and meta-analysis of case-control studies were conducted following PRISMA guidelines. Literature from PubMed/MEDLINE, Scopus, Cochrane, and Embase (2000 onward) was screened. Random-effects models were used in R, with subgroup analyses by geography, case definition, diagnostics, and matching criteria.

**Results:**

Out of 2941 records, 26 case-control studies (11 706 participants) met inclusion criteria. Most studies used molecular diagnostics (n = 23). A significant association was found between CT infection and male infertility (odds ratio [OR] 3.68 [95% CI 2.24–6.02]), with substantial heterogeneity (*I*^2^ = 65%). Age-matched studies showed higher effect sizes (OR 6.77), and publication bias was detected (trimmed OR 2.75).

**Conclusions:**

While findings suggest that CT infection may impair male fertility, confounding, bias, and the lack of geographical representativeness limit inference. High-quality, large-scale prospective studies are needed to confirm causality and guide targeted interventions.

Infertility, defined as the failure to conceive after 1 year or longer of regular unprotected sexual intercourse [1], is estimated to affect up to 12% of couples in the general population, impacting millions of individuals worldwide [2]. As a global public health issue, infertility is associated with poor health outcomes in terms of physical and mental well-being and can have detrimental societal implications, including stigma and financial hardship [2]. As such, it represents an important component of sexual and reproductive health and rights [3], necessitating further research to inform policies and interventions. Studies have shown that male factors may contribute to 50% of infertility cases [4]. Although data are scarce, an estimated 15% of those could be attributed to infections, including sexually transmitted infections (STIs) [4].

*Chlamydia trachomatis* (CT) is the most common bacterial STI worldwide [5], with increasing trends observed in the last decades [6, 7]. The high prevalence of CT infection makes it a significant public health concern, with a recent meta-analysis (MA) reporting a global prevalence of genital CT infection at 2.9% (95% confidence interval [CI], 2.4%–3.5%) among the general population [8]. CT disproportionately affects women [9], leading to severe reproductive health issues [10]. Among these, infertility is the most notable sequela, resulting from untreated or inadequately treated infections that cause damage to the reproductive organs. Although CT's impact on women is well-documented, the literature on CT in males is relatively limited. Nonetheless, despite the narrower scope of studies in men, it is recognized that one of the crucial sequelae of CT in males is infertility [11].

Several mechanisms, including epididymal scarring and obstruction, have been proposed to explain how CT infection may affect semen quality [12–15]. Despite these biological insights, few epidemiological studies have quantified this association at the population level. Understanding the consequences of CT infection in males is essential to developing effective public health strategies for control and preventative interventions.

Given the limited information concerning the impact of CT infection on male fertility and the variability in findings across different studies, we performed a systematic literature review (SLR) and MA to critically assess and synthesize the current evidence.

## METHODS

The findings of the present SLR and MA were reported in accordance with the requirements outlined in the “Preferred Reporting Items for Systematic Reviews and Meta-Analysis” checklist [16].

### Literature Search

A search string consisting of keywords related to 3 major components (CT, males, and infertility) was utilized. Wild-card and Medical Subject Headings options were leveraged. The search string was devised with the help of a librarian, after familiarization with the existing body of literature and incorporating all existing synonyms related to CT, infertility, sperm phenotypes, and semen anomalies. The literature search was conducted independently by N. L. B. and a research librarian in 4 major scholarly electronic databases: PubMed/MEDLINE, Embase, Scopus, and the Cochrane Library. No language filters were applied, while a time filter was implemented to ensure that CT ascertainment was reliable and consistent, with 2000 as a time cutoff for the year of publication through 5 July 2024 (Supplementary Table 1).

### Inclusion and Exclusion Criteria

Inclusion/exclusion criteria were devised according to the “Population/Exposure/Control/Outcome/Study design” (PECOS) mnemonic: in a (P)opulation of infertile males, the (E)xposure was to CT, and the (C)ontrol was apparently healthy males. The (O)utcome was infertility, whose association with CT infection was quantified as odds ratios (ORs). The (S)tudy design was case-control studies. We reported only on case–control studies to maintain methodological comparability. Although prospective cohort and time-to-event designs (eg, longitudinal or registry-based studies) would provide stronger temporal and causal inference, no such studies were identified in our search.

Articles focusing on animals or using *in vitro* models (P) were excluded, as well as those recruiting females only or recruiting males but not providing data disaggregated for sex/gender. Studies with individuals not exposed to CT (E) were not deemed eligible. Studies that did not include apparently healthy males as controls (C) and did not focus on infertility (O) were excluded. Finally, case reports, reviews, and cross-sectional studies (S) were excluded. Existing SLRs and MAs [17–21] were scanned to reduce the risk of missing potentially relevant papers, but were not retained.

### Data Extraction

Data encompassing study characteristics such as publication and study year, country, sample size, and participant demographics were independently extracted by N. L. B. and V. B. C. Detailed information on inclusion/exclusion criteria was collected to assess the comparability of the studies. Diagnostic techniques used to detect CT, such as polymerase chain reaction (PCR), enzyme-linked immunosorbent assay (ELISA), and cell culturing, were noted alongside the types of biological samples analyzed. Key outcomes related to semen quality, such as sperm concentration, motility, morphology, vitality, and leukocyte counts, were extracted. Additionally, the prevalence of CT infection among infertile and fertile males, as well as any reported immunological responses, was documented (Supplementary Table 2).

### Study Quality Assessment

Methodological quality and robustness of the studies included were assessed independently by 2 of the authors (N. L. B. and V. B. C), both informally and formally, noting the strengths and limitations of the studies and using the Joanna Briggs Institute (JBI) tool for appraising case-control studies (the “JBI Checklist for Case-Control Studies”) [22]. This 10-item checklist evaluates several critical aspects to ensure the robustness of the study design and the validity of the findings. First, it assesses whether the groups were comparable other than the presence or absence of the disease, ensuring that any differences in outcomes are likely due to the exposure rather than other variables. It examines if cases and controls were matched appropriately and whether the same criteria were used for their identification to minimize selection bias. The tool checks if the exposure was measured in a standardized, reliable way, and whether this measurement was consistent for both cases and controls. Further, it assesses whether confounding factors were noted, with strategies to address them, to rule out alternative explanations for the findings. It checks if outcomes were assessed in a valid and reliable manner for both cases and controls, with the exposure period sufficiently long to capture meaningful data. Lastly, the tool verifies if appropriate statistical analyses were used, ensuring that the conclusions drawn are statistically sound.

### Meta-analysis

We considered infertility as the primary outcome, defined based on the World Health Organization (WHO) criteria [1], as well as laboratory and clinical assessments of sperm quality. Given the variability in how infertility was classified across studies, and to account for potential heterogeneity, we conducted subgroup analyses based on case definitions of infertility, considering also diagnostic ascertainment of infertility. For exposure, we considered multiple approaches to defining CT infection status, including self-reported history of infection, as documented in medical questionnaires or interviews, clinical diagnosis based on physician assessment and medical records, serological evidence of infection, indicating past or current exposure to the pathogen, and Nucleic Acid Amplification Test (NAAT) results, which provide direct detection of pathogen-specific DNA.

We compared cases of infertility between exposed and non-exposed groups, estimating the OR to assess the association. In particular, since the OR is not normally distributed, we applied a log transformation. Specifically, the variance of the log OR was estimated using the inverse sum of the 4 cell frequencies in a 2 × 2 contingency table. The standard error (SE) of the log OR was derived from this variance estimate, and the CIs were constructed using the normal approximation and exponentiated to provide the 95% CI on the original scale. The overall OR was computed by pooling together the ORs of the individual studies using the inverse variance method, which allows for the weighting of studies according to their precision, ensuring that the final pooled estimate is as accurate and unbiased as possible and, thus, enhancing the overall quality and interpretability of meta-analytical findings [23]. For studies with a zero cell count, a continuity correction was applied, whereby 0.5 was added to all cell frequencies when calculating the OR.

Heterogeneity was assessed using the *I*² statistic, with a value of 0% indicating no observed heterogeneity and higher values indicating increasing levels of heterogeneity. Heterogeneity was classified as low, moderate, or high based on *I*² values of 25%, 50%, and 75%, respectively. To account for heterogeneity, a random-effects model was employed.

Meta-regressions according to the publication year, participants’ age, and sample size were carried out. Subgroup analyses based on study country, diagnostic methods, use of age-matched case-controls, clinical confirmation of infertility, and whether studies had *a priori* power assessments were conducted to further explore potential sources of heterogeneity among studies.

The presence of potential publication biases was investigated by visually inspecting the funnel plot and conducting statistical tests, including Egger's regression test for funnel plot asymmetry and the trim-and-fill method. Egger's test assesses small-study effects by regressing the standardized ES on their SEs. The trim-and-fill method was applied to estimate the number of missing studies and adjust the overall ES accordingly.

The meta-analytical computation was carried out in the open-source R computational environment (version 4.2.3, The R Foundation for Statistical Computing Platform), using the “*metabin*” package [24].

## RESULTS

### Systematic Review: Characteristics of the Studies Included

**Figure 1.**
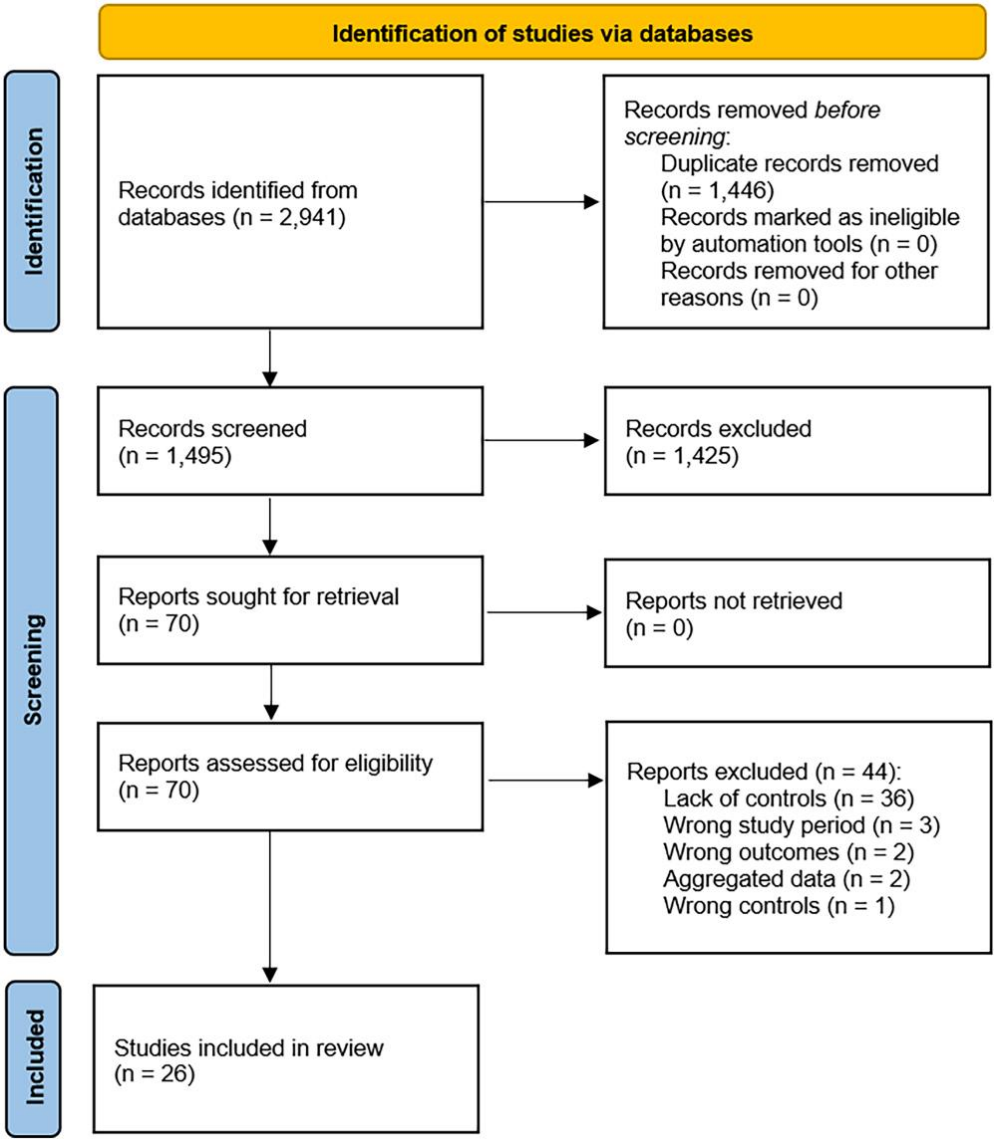
Pictorial flowchart of the study identification process adopted in the present systematic review and meta-analysis.

The search identified 2941 items (namely, 626 from PubMed/MEDLINE, 1100 from Scopus, 38 from Cochrane, and 1177 from Embase) before deduplication and 1495 unique items after deduplication, after the removal of 1446 duplicates. Based on the title and/or abstract, 1425 items were removed. Seventy items were accessed in full text, of which 44 were excluded with reason (n = 36, because of lack of controls; n = 3, because of wrong study period; n = 2, because of wrong outcomes; n = 2, because it was not possible to disaggregate/stratify data; n = 1, because of wrong controls). Finally, 26 studies [25–50] were retained (Figure 1), published between 2003 [25] and 2024 [50].

**Figure 2.**
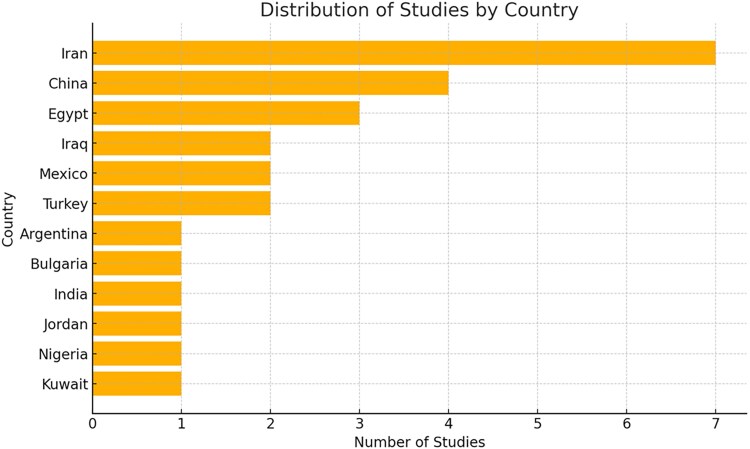
Distribution of studies included in the present systematic review and meta-analysis by country.

The studies were conducted across various countries (Figure 2), with 7 originating from Iran [26, 28, 29, 36, 38, 39, 43], 4 from China [25, 27, 41, 50], and 3 from Egypt [31, 32, 48]. Additionally, 2 studies were based in Iraq [37, 40], 2 in Mexico [33, 35], and 1 in Turkey [46, 47]. Single studies were conducted in Argentina [34], Bulgaria [49], India [30], Jordan [42], Nigeria [44], and Kuwait [45]. Sample sizes from the various studies included in the present review varied from 76 [40] to 3950 [34]. The studies totaled 11 706 participants (7934 cases and 3772 controls).

Most studies used NAATs (n = 23) [25–43, 45, 47–49] with 5 [30–32, 43 , 48] also incorporating immunoassays and 1 study [49] cell culture. A smaller subset (n = 3) [44, 46, 50] relied on immunoassays to ascertain CT exposure. Additionally, 1 study [50] coupled immunoassays with immunochromatography, while another [46] employed cell culture alongside immunoassays and immunochromatography. No study relied on self-reported exposure.

Fourteen studies [26, 28, 29, 31, 32, 34, 36–40, 45, 47, 48] analyzed seminal fluid, while only 1 study [44] collected blood and 1 further study [43] analyzed both blood and seminal fluid. Two studies [33, 35] analyzed urine samples, with 3 further studies [27, 41, 49] collecting urethral swabs. In 1 study [46], urethral swabs were analyzed alongside blood and alongside seminal fluids in another study [25]. One study [42] collected urine alongside seminal fluid. Finally, 2 studies [29, 50] collected a variety of biological specimens, such as urethral swabs, urine, and seminal fluid [50], and urine, seminal fluid, and serum [30] (Table 1 and Supplementary Table 2).

**Table 1.** Main Characteristics of the Studies Included in the Present Systematic Review and Meta-Analysis of the Association Between *Chlamydia trachomatis* and Male Infertility

Study	Country	Infertility Case Definition	Sperm Sample Collection	Sample Size	Sample Power Analysis	Age	Age Matching	Cases	Controls	Cases With CT	Controls With CT	Diagnostic Method	Biological Sample
Zeng et al [25]	China	Clinician confirmed	NS	611	No	31.36 ± 4.93y	Unclear	456	155	10	9	PCR	Genital secretions and fluids
Ahmadi et al [26]	Iran	Clinician confirmed	2–3 d	100	Yes	31 ± 4.1 y	Yes	50	50	5	0	PCR	Seminal fluid
Li et al [27]	China	Clinician and lab confirmed	2–7 d	883	No	33.0 ± 5.4 y in the infertility group, 34.6 ± 5.1y in the control group	Yes	393	490	24	3	PCR	Uro-genital swabs
Dehghan et al [28]	Iran	WHO-based	2–7 d	130	No	35.2 ± 6.8y	Unclear	65	65	6	2	PCR	Seminal fluid
Haidari Pebdeni et al [29]	Iran	WHO-based	2–7 d	200	Yes	35.33 ± 7.38 y in the infertility group, 34.29 ± 7.3 y in the fertility group	Yes	100	100	9	0	PCR	Seminal fluid
Naik et al [30]	India	NS	NS	139	No	35 y	Unclear	79	60	1	0	PCR, ELISA	Urine, seminal fluid, serum
El-Din et al [31]	Egypt	WHO-based, clinician confirmed	3–5 d	265	No	38 ± 2 y in the infertility group, 29 ± 1 y in the fertility group	No	200	65	15	0	PCR, ELISA	Seminal fluid
EzzEl-Din et al [32]	Egypt	Lab confirmed	3–5 d	275	No	35.30 ± 5.90 y in the infertility group; 36.20 ± 5.74 y in the control group	Yes	250	25	60	0	PCR, ELISA	Seminal fluid
López-Hurtado et al [33]	Mexico	NS	NS	659	No	32.6 y	Unclear	287	372	43	6	PCR	Urine
Paira et al [34]	Argentina	WHO-based, clinician confirmed	2–7 d	3950	No	18–60 y	Unclear	3610	340	208	6	PCR	Seminal fluid
López-Hurtado et al [35]	Mexico	Lab and imaging confirmed	NS	668	No	20–52 y	Unclear	269	399	25	33	PCR	Urine
Motamedifar et al [36]	Iran	NS	3 d	350	No	36 ± 7.0 y in the infertility group, 36 ± 6.9 y in the fertility group	Yes	200	150	25	2	PCR	Seminal fluid
Hassan et al [37]	Iraq	WHO-based, clinician confirmed	NS	200	No	28.92 ± 5.9 y in the infertility group, 27.73 ± 3.9 y in the fertility group	Yes	100	100	17	1	PCR	Seminal fluid
Moosavian et al [38]	Iran	WHO-based, clinician-confirmed	At least 48 h (4–5 d)	100	Yes	31.4y	Unclear	50	50	5	0	PCR	Seminal fluid
Ahmadi et al [39]	Iran	Lab confirmed	3–7 d	330	No	34.3 ± 0.4 y in infertility group, 33.6 ± 0.4 y in fertility group	Yes	165	165	7	1	PCR	Seminal fluid
Ali et al [40]	Iraq	WHO-based, lab-confirmed	NS	76	No	32.28 ± 6.88 y in the infertility group, 34.07 ± 6.52 y in the fertility group	Yes	63	13	11	0	PCR	Seminal fluid
Liu et al [41]	China	WHO-based	3–5 d	1236	No	NA	Unclear	621	615	16	14	PCR	Urethral swabs
Abusarah et al [42]	Jordan	Lab confirmed	NS	163	No	33 ± 8.07 y in the infertility group, 32 ± 6.74 y in the fertility group	Yes	93	70	4	1	PCR	Urine, seminal fluid
Noruziyan et al [43]	Iran	WHO-based	NS	186	No	32.8 ± 6.3 y in the infertility group, 36.7 ± 6.5 y in the fertility group	No	93	93	18	7	PCR, ELISA	Blood, seminal fluid
Osazuwa et al [44]	Nigeria	Lab confirmed	3–5 d	255	No	20–29 y	Unclear	215	40	42	2	Immunoassay	Blood
Al-Sweih et al [45]	Kuwait	WHO-based, lab confirmed	5 d	315	No	NS	Unclear	127	188	4	5	PCR	Seminal fluid
Günyeli et al [46]	Turkey	NS	3 d	106	No	30.43 ± 5.58 y for infertility group, 37.66 ± 7.02 y in the fertility group	No	53	53	2	1	ELISA, immunochromatography, cell culture	Blood, urethral swabs
Çalışkan et al [47]	Turkey	Lab confirmed	3–4 d	175	No	19–47y	Yes	144	31	12	3	PCR	Seminal fluid
El Feky et al [48]	Egypt	Lab confirmed	3–5 d	100	No	30.8 ± 5.93 y in the infertility group, 33.08 ± 7.72 y in the fertility group	Yes	75	25	23	0	PCR, ELISA	Seminal fluid
Ouzounova-Raykova et al [49]	Bulgaria	Clinician and lab confirmed	3–4 d (from 48 h to 7 d)	100	No	31 y in the infertility group, 30 y in the fertility group	Yes	60	40	5	1	PCR, cell culture	Urethral swabs
Liu and Zhu, 2003 [50]	China	Clinician confirmed	At least 24h	134	No	29.7 ± 3.4 y in the infertility group, 28.6 ± 3.4 y in the fertility group	Yes	116	18	30	0	ELISA, immunochromatography	Urethral swab, urine, seminal fluid

### Systematic Review: An Overview of the Major Findings of the Studies Included

Nine studies [25, 30, 41, 42, 45–47, 49, 50] found no significant association between CT infection and male infertility. Among these studies, Abusarah et al [42] detected a higher CT prevalence among younger infertile individuals but could not establish a clear, significant link with infertility. Liu and Zhu [50] reported that among infertile men, antibody prevalence was higher in those with abnormal sperm density but lacked statistical significance. Antibody to CT was also unrelated to sperm motility. Finally, other studies [41, 46] reported no significant differences in CT prevalence and semen parameters.

Eight studies [26, 28, 31, 32, 35, 38–40] reported mixed findings. While they failed to find a significant association between CT infection and infertility, they observed effects on semen parameters. Moosavian et al [38] found that, although the CT prevalence rate in infertile subjects was not significantly different from that of fertile individuals, Western blot and Terminal deoxynucleotidyl transferase dUTP Nick-End Labeling assay results showed significantly increased caspase-3 activation and DNA fragmentation in CT-exposed infertile men. In the study by Ali and Al-Kazaz [40], CT-exposed infertile men exhibited significantly lower sperm count, motility, and morphology. Similarly, Dehghan et al [28] reported a strong correlation between CT infection and impaired semen parameters. In the study by Ahmadi et al [26], men with infertility showed reduced sperm motility, a finding replicated by El-Din et al [31] and by López-Hurtado et al [35]. EzzEl-Din et al [32] demonstrated that males with CT-infection, particularly those with leukocytospermia, exhibited impaired sperm concentration, motility, and viability, which closely correlated with elevated reactive oxygen species levels and increased sperm DNA fragmentation. Finally, Ahmadi et al [39] demonstrated that antibiotic treatment improved semen quality and fertility outcomes in CT-positive infertile men.

Nine studies [27, 29, 33, 34, 36, 37, 43, 44, 48] were able to establish a significant association between CT infection and male infertility. Paira et al [34] reported a higher CT prevalence in patients with infertility than in controls, particularly in those under 25. Heidari Pebdeni et al [29] detected a strong association between CT infection and impaired sperm quality, a finding confirmed by Hassan et al [37], who documented altered sperm motility and morphology in infertile individuals with CT infection. Furthermore, López-Hurtado et al [33] linked CT infection to sperm abnormalities such as teratozoospermia and azoospermia. Osazuwa et al [44] found a higher CT seroprevalence among infertile men, especially in younger individuals, and in patients with oligozoospermia and azoospermia. Motamedifar et al [36] identified CT as the most frequently detected pathogen in infertile men with bacteriospermia. However, El Feky et al [48] investigated CT infection in infertile men with leukocytospermia but found no significant impact on semen parameters. IgA antibodies were present in 26.7% of patients, plasmid DNA in 30.7%, and CT elementary bodies in 46.6%. Despite these findings, routine semen parameters remained unaffected.

### Quality Assessment

The overall quality of the included studies was low, with a median score of 45%, ranging from 30% [30, 33, 46] to 70% [39] (Supplementary Table 3). Thirteen studies [26, 27, 29, 32, 36, 37 , 39, 40, 42, 47–50] performed age-based matching, while in 10 [23, 28, 30, 33–35, 38, 41, 44, 45] and 3 [31, 43, 46] studies, the matching was unclear or not performed, respectively. Only 3 studies [26, 29, 38] performed *a priori* sample size power analysis.

In 4 studies [28, 29, 41, 43] infertility was defined according to the WHO criteria. Another 3 studies [25, 26, 50] used clinician-confirmed case ascertainment. Similarly, 4 studies [31, 34, 37, 38] applied a combination of clinical assessment and WHO-based criteria. Two studies [27, 49] required both clinical confirmation and laboratory testing to establish infertility. In contrast, 6 studies [32, 39, 42, 44, 47, 48] diagnosed infertility based on abnormal semen parameters confirmed through laboratory analysis. One study [35] refined infertility diagnosis by integrating laboratory findings with imaging-based assessments, while another 2 studies [40, 41] combined WHO criteria with laboratory test results. Lastly, in 4 studies [30, 31, 36, 46], the specific criteria used to define infertility were not reported.

Out of the 19 studies [25, 26, 28–32, 34, 36–40, 42–44, 47, 48, 50] that collected seminal fluid, 8 studies [25, 30, 37, 40, 42, 43] did not specify when sperm samples were collected. When reported, the period of sexual abstinence prior to sample collection ranged from 1 to 7 days.

The exclusion criteria varied across studies but typically encompassed factors that could confound the association between infertility and the investigated parameters. These included (1) pre-existing genitourinary conditions, (2) prior vasectomy or sterilization procedures, (3) genital tract infections/STIs, (4) testicular tumors or previous testicular surgery, (5) endocrine disorders, (6) medical and lifestyle factors, like use of antibiotics from 1 week to 1 month before sampling, history of chemotherapy/radiotherapy, heavy alcohol consumption, smoking, or drug use, exposure to environmental toxins, radiation, or chemicals with documented reproductive toxicity, obesity, diabetes, and hypertension. Furthermore, some studies excluded participants with prior fertility treatments or assisted reproductive technology use. The presence of known female-related infertility factors was also an exclusion criterion in some studies to isolate male-specific infertility. Other exclusion criteria included psychiatric conditions or emotional distress affecting reproductive behavior, genetic disorders, or chromosomal anomalies. However, only 4 studies comprehensively identified confounding factors [28, 39, 41, 49]. None of the studies performed multivariable adjustment, leaving the evidence vulnerable to confounding, including unmeasured factors.

### Meta-analysis

**Figure 3.**
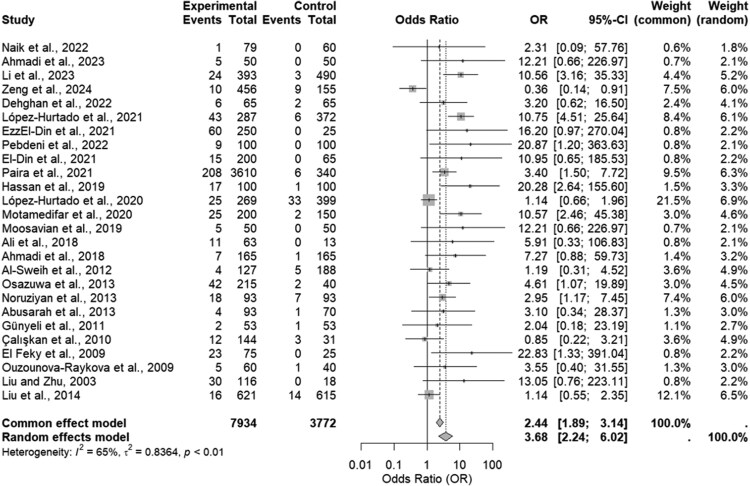
Forest plot of the association between *Chlamydia trachomatis* infection and male infertility.

The overall ES for the random-effects model (moderate heterogeneity, I²=65%, *P* < .01) was 3.68 (95% CI 2.24–6.02), indicating a strong association between CT infection and male infertility (Figure 3). Sensitivity analysis confirmed the stability of the findings (Supplementary Figure 1).

Meta-regressions (Supplementary Figures 2–4) did not show any impact of publication year (*P* = .600), age (*P* = .451), or sample size (*P* = .563). Subgroup analysis results are shown in Supplementary Figures 5–8. Study country (*P* = .211) and the diagnostic method employed (*P* = .922) had no impact, as well as clinical confirmation of infertility (*P* = .351). Age-based matching in choosing cases and controls had a marginally significant impact (*P* = .047): properly matched studies reported a higher ES (6.77 [95% CI 3.65–12.55]) than studies with unclear (2.13 [95% CI 1.05–4.33]) or no matching (3.16 [95% CI 1.38–7.23]). Similarly, the impact of conducting a priori sample size power analysis was found to be borderline (*P* = .098): the studies that performed such an analysis reported a higher ES, even though with a wide CI (14.68 [95% CI 2.75–78.33]) than those that did not carry out such an analysis (3.35 [95% CI 2.01–5.58]).

**Figure 4.**
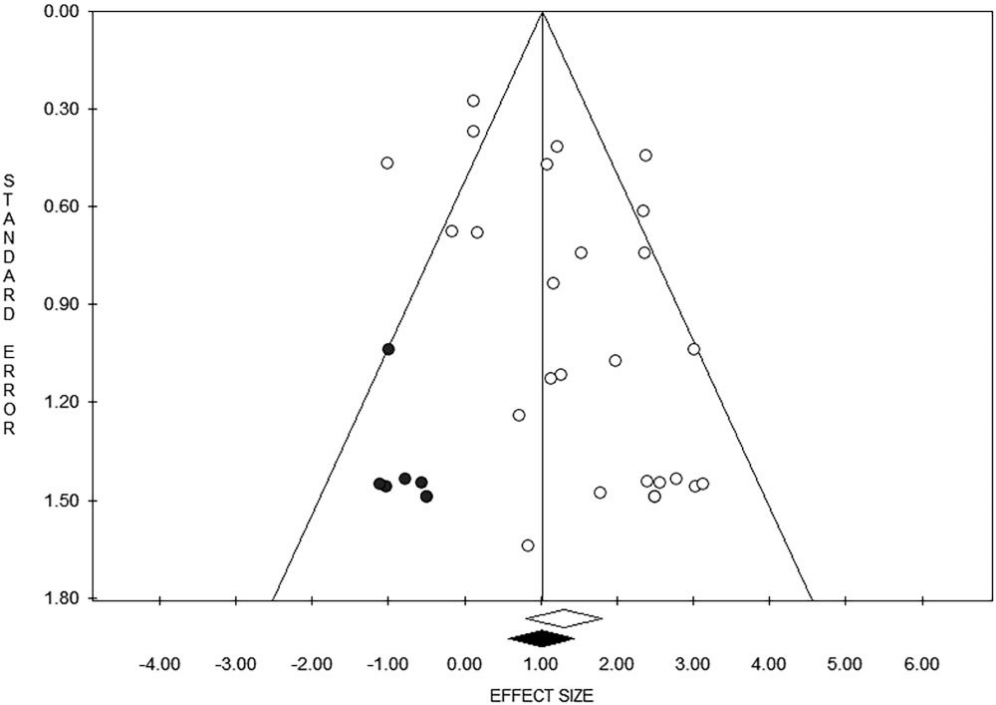
Funnel plot, showing evidence of publication bias computed by the trim-and-fill analysis (trimmed effect size computed at 2.75 [95% CI 1.74–4.35]).

The visual inspection of the funnel plot (Figure 4) and Egger's test (Supplementary Figure 9) showed evidence of publication bias. The trimmed ES was computed at 2.75 [95% CI 1.74–4.35] (Figure 4).

## DISCUSSION

Despite the well-established impact of CT on female reproductive health, its implications for male fertility have remained relatively underexplored. Biologically, CT infection may impair sperm function through multiple mechanisms, including sperm apoptosis triggered by chlamydial lipopolysaccharide, persistent infection leading to scarring of the ejaculatory ducts, and the loss of stereocilia. Additionally, epithelial cell damage in the spermatogenic process may further impair sperm quality [12–15]. Nonetheless, the existing literature presents conflicting findings. Compared to existing SLRs and MAs [17, 21], our SLR/MA adopts a more methodologically robust approach by incorporating meta-regressions, subgroup analyses, and critical quality assessments. Our results suggest a significant association between CT infection and male infertility, reinforcing the need for greater clinical attention to CT management in males.

However, the observed heterogeneity across studies highlights the complexity of this relationship. On the one hand, meta-regressions and subgroup analyses suggested that the association between CT infection and male infertility was relatively robust across different contexts and methodological approaches. On the other hand, most studies employed small sample sizes, thus constraining statistical power, which can explain the width of the 95% CI of several OR estimates. Furthermore, inclusion/exclusion criteria varied widely [28, 39, 41, 49], leading to inconsistencies in participant selection. The lack of multivariable adjustment raises the possibility that the observed associations between CT infection and impaired semen parameters may be inflated. Although, as previously stated, our subgroup analyses and meta-regressions suggest that the association is broadly consistent across settings, the lack of confounder adjustments, and other methodological inconsistencies prevent firm causal inference.

Moreover, study quality had an impact on the pooled OR, with studies that employed age matching when selecting cases and controls reporting significantly higher ESs [26, 27, 29, 32, 36, 37, 39, 40, 42, 47–50]. Additionally, studies that conducted *a priori* sample size power analyses [26, 29, 38] reported a stronger association, although this finding did not reach significance thresholds. Another key limitation of the current body of evidence is the finding that, while publication bias may influence the magnitude of the association, it does not entirely account for the observed relationship between CT infection and male infertility. This underscores the importance of future prospective studies with rigorous methodology and transparent reporting to mitigate the influence of selective publication.

Also, all the studies included were conducted in low- and middle-income countries, with no studies originating from the Global North. This geographic imbalance raises concerns regarding the generalizability of the findings, given differences in CT epidemiology, sexual health services, population structure, and infertility etiologies. A WHO report on population-based infertility prevalence estimates from 1990 to 2021 suggested that the Americas, Europe, and the Western Pacific region have higher infertility prevalence than other regions in the globe [51]. This report, however, covered a wider research period and any cause of infertility in both males and females, not directly comparable to our study. Nonetheless, the report raised important knowledge gaps in male reproductive epidemiology, considering the challenges related to exposure measurements, representativeness of different countries, limited studies done in males, lack of reliable biomarkers, and the variations in infertility definition and inclusion/exclusion criteria.

Despite these limitations, our findings have important public health implications. Given the high global prevalence of CT infection and its established role in female infertility [9, 10], early detection and treatment of CT infection may be justified. Currently, CT screening is routine only in a few countries and focuses on pregnant women, young women <25, and high-risk groups (eg, men who have sex with men and sex workers) [6, 7]. For males, understanding and quantifying the association of CT infection and infertility could guide prioritization of resources and new interventions to improve patient management. Further research is warranted to refine our understanding of this association and assess the long-term reproductive health outcomes of CT-infection in males and evaluate the effectiveness of targeted interventions, including the potential benefits of future vaccines.

## Supplementary Material

ofaf782_Supplementary_Data

## Supplementary Data

Supplementary materials are available at *Open Forum Infectious Diseases* online. Consisting of data provided by the authors to benefit the reader, the posted materials are not copyedited and are the sole responsibility of the authors, so questions or comments should be addressed to the corresponding author.

## References

[1] Zegers-Hochschild F, Adamson GD, Dyer S, et al The international glossary on infertility and fertility care, 2017. Fertil Steril 2017; 108:393–406. 28760517 10.1016/j.fertnstert.2017.06.005

[2] Inhorn MC, Patrizio P. Infertility around the globe: new thinking on gender, reproductive technologies and global movements in the 21st century. Hum Reprod Update 2015; 21:411–26. 25801630 10.1093/humupd/dmv016

[3] Ghebreyesus TA, Allotey P, Narasimhan M. Advancing the “sexual” in sexual and reproductive health and rights: a global health, gender equality and human rights imperative. Bull World Health Organ 2024; 102:77–8. 38164333 10.2471/BLT.23.291227 PMC10753275

[4] Goulart ACX, Farnezi HCM, França JPBM, Santos AD, Ramos MG, Penna MLF. HIV, HPV and Chlamydia trachomatis: impacts on male fertility. JBRA Assist Reprod 2020; 24:492–7. 32496735 10.5935/1518-0557.20200020 PMC7558888

[5] Hocking JS, Geisler WM, Kong FYS. Update on the epidemiology, screening, and management of Chlamydia trachomatis infection. Infect Dis Clin North Am 2023; 37:267–88. 37005162 10.1016/j.idc.2023.02.007

[6] Du M, Yan W, Jing W, et al Increasing incidence rates of sexually transmitted infections from 2010 to 2019: an analysis of temporal trends by geographical regions and age groups from the 2019 Global Burden of Disease Study. BMC Infect Dis 2022; 22:574. 35754034 10.1186/s12879-022-07544-7 PMC9233762

[7] Huai P, Li F, Chu T, Liu D, Liu J, Zhang F. Prevalence of genital Chlamydia trachomatis infection in the general population: a meta-analysis. BMC Infect Dis 2020; 20:589. 32770958 10.1186/s12879-020-05307-w PMC7414538

[8] Van Gerwen OT, Muzny CA, Marrazzo JM. Sexually transmitted infections and female reproductive health. Nat Microbiol 2022; 7:1116–26. 35918418 10.1038/s41564-022-01177-x PMC9362696

[9] Hoenderboom BM, van Benthem BHB, van Bergen JEAM, et al Relation between Chlamydia trachomatis infection and pelvic inflammatory disease, ectopic pregnancy and tubal factor infertility in a Dutch cohort of women previously tested for chlamydia in a chlamydia screening trial. Sex Transm Infect 2019; 95:300–6. 30606817 10.1136/sextrans-2018-053778 PMC6585279

[10] Cunningham KA, Beagley KW. Male genital tract chlamydial infection: implications for pathology and infertility. Biol Reprod 2008; 79:180–9. 18480466 10.1095/biolreprod.108.067835

[11] Eley A, Hosseinzadeh S, Hakimi H, Geary I, Pacey AA. Apoptosis of ejaculated human sperm is induced by co-incubation with Chlamydia trachomatis lipopolysaccharide. Hum Reprod 2005; 20:2601–7. 15905291 10.1093/humrep/dei082

[12] Zhou H, Wu S, Tang X, et al Chlamydia trachomatis infection in the genital tract is associated with inflammation and hypospermia in the infertile male of China. Asian J Androl 2022; 24:56–61. 34145079 10.4103/aja.aja_54_21 PMC8788609

[13] Liu KS, Mao XD, Pan F, An RF. Effect and mechanisms of reproductive tract infection on oxidative stress parameters, sperm DNA fragmentation, and semen quality in infertile males. Reprod Biol Endocrinol 2021; 19:97. 34183027 10.1186/s12958-021-00781-6 PMC8237428

[14] Gallegos G, Ramos B, Santiso R, Goyanes V, Gosálvez J, Fernández JL. Sperm DNA fragmentation in infertile men with genitourinary infection by Chlamydia trachomatis and Mycoplasma. Fertil Steril 2008; 90:328–34. 17953955 10.1016/j.fertnstert.2007.06.035

[15] Joki-Korpela P, Sahrakorpi N, Halttunen M, Surcel HM, Paavonen J, Tiitinen A. The role of Chlamydia trachomatis infection in male infertility. Fertil Steril 2009; 91:1448–50. 18706556 10.1016/j.fertnstert.2008.06.051

[16] Page MJ, McKenzie JE, Bossuyt PM, et al The PRISMA 2020 statement: an updated guideline for reporting systematic reviews. BMJ 2021; 372:n71. 33782057 10.1136/bmj.n71 PMC8005924

[17] Keikha M, Hosseininasab-Nodoushan SA, Sahebkar A. Association between Chlamydia trachomatis infection and male infertility: a systematic review and meta-analysis. Mini Rev Med Chem 2023; 23:746–55. 36043714 10.2174/1389557522666220827160659

[18] Henkel R . Long-term consequences of sexually transmitted infections on men's sexual function: a systematic review. Arab J Urol 2021; 19:411–8. 34552793 10.1080/2090598X.2021.1942414 PMC8451632

[19] Farahani L, Tharakan T, Yap T, Ramsay JW, Jayasena CN, Minhas S. The semen microbiome and its impact on sperm function and male fertility: a systematic review and meta-analysis. Andrology 2021; 9:115–44. 32794312 10.1111/andr.12886

[20] Fode M, Fusco F, Lipshultz L, Weidner W. Sexually transmitted disease and male infertility: a systematic review. Eur Urol Focus 2016; 2:383–93. 28723470 10.1016/j.euf.2016.08.002

[21] Ahmadi MH, Mirsalehian A, Bahador A. Association of Chlamydia trachomatis with infertility and clinical manifestations: a systematic review and meta-analysis of case-control studies. Infect Dis (Lond) 2016; 48:517–23. 27064452 10.3109/23744235.2016.1160421

[22] The Joanna Briggs Institute Critical Appraisal tools for use in JBI systematic reviews checklist for case control studies . Available at: https://jbi.global/sites/default/files/2019-05/JBI_Critical_Appraisal-Checklist_for_Case_Control_Studies2017_0.pdf.

[23] Borenstein M, Hedges L, Higgins J, Rothstein H. Generality of the basic inverse-variance method. In: Introduction to meta-analysis. Chichester: John Wiley & Sons, Ltd.: 2009, 311–319.

[24] meta (version 6.5–0). metabin: Meta-analysis of binary outcome data. Available at: https://www.rdocumentation.org/packages/meta/versions/6.5-0/topics/metabin.

[25] Zeng J, Wu T, Wang L, Yu L, Lin H, Chen Z. Characteristics of reproductive tract infections caused by common pathogens among the outpatients of reproductive medicine center in Putian: retrospective study. BMC Infect Dis 2024; 24:315. 38486167 10.1186/s12879-024-09180-9 PMC10941379

[26] Ahmadi K, Moosavian M, Mardaneh J, Pouresmaeil O, Afzali M. Prevalence of Chlamydia trachomatis, Ureaplasma parvum and Mycoplasma genitalium in Infertile Couples and the Effect on Semen Parameters. Ethiop J Health Sci 2023; 33:133–42. 36890937 10.4314/ejhs.v33i1.17 PMC9987280

[27] Li M, Hu CX, Lu XL, et al [Effects of urogenital mycoplasma and chlamydial infections on male fertility]. Zhonghua Nan Ke Xue 2023; 29:639–44. 38619413

[28] Dehghan A, Pourmand MR, Salimi V, et al The effects of Chlamydia trachomatis, Mycoplasma hominis, and Ureaplasma urealyticum loads on semen quality: detection and quantitative analysis. Microb Pathog 2022; 169:105676. 35820579 10.1016/j.micpath.2022.105676

[29] Heidari Pebdeni P, Saffari F, Reza Mirshekari T, Ashourzadeh S, Taheri Soodejani M, Ahmadrajabi R. Bacteriospermia and its association with seminal fluid parameters and infertility in infertile men, Kerman, Iran: a cross-sectional study. Int J Reprod Biomed 2022; 20:202–12. 35571500 10.18502/ijrm.v20i3.10712 PMC9099366

[30] Naik KV, Mishra A, Panda S, et al Seropositivity of Chlamydia trachomatis & Toxoplasma gondii among male partners of infertile couples in Odisha, India: a facility-based exploratory study. Indian J Med Res 2022; 156(4&5):681–4. 36926786 10.4103/ijmr.IJMR_83_21 PMC10231736

[31] Nasr El-Din A, Sorour H, Fattouh M, Abu El-Hamd M. Evaluation of the role of Chlamydia trachomatis in primary male infertility. Int J Clin Pract 2021; 75:e14702. 34378266 10.1111/ijcp.14702

[32] EzzEl-Din AM, Gaber HD, Kamal DT. Chlamydia trachomatis infection: its relation to semen parameters and sperm DNA integrity. Egypt J Immunol 2021; 28:290–8. 34882378

[33] López-Hurtado M, Escarcega-Tame MA, Escobedo-Guerra MR, de Haro-Cruz MJ, Guerra-Infante FM. Identification of Chlamydia trachomatis genotypes in Mexican men with infertile women as sexual partners. Enferm Infecc Microbiol Clin (Engl Ed) 2022; 40:353–8. 35906030 10.1016/j.eimce.2021.02.012

[34] Paira DA, Molina G, Tissera AD, Olivera C, Molina RI, Motrich RD. Results from a large cross-sectional study assessing Chlamydia trachomatis, Ureaplasma spp. and Mycoplasma hominis urogenital infections in patients with primary infertility. Sci Rep 2021; 11:13655. 34211075 10.1038/s41598-021-93318-1 PMC8249471

[35] López-Hurtado M, Flores-Salazar VR, Gutierréz-Trujillo R, Guerra-Infante FM. Prevalence, concordance and reproductive sequelae after Chlamydia trachomatis infection in Mexican infertile couples. Andrologia 2020; 52:e13772. 32722871 10.1111/and.13772

[36] Motamedifar M, Malekzadegan Y, Namdari P, Dehghani B, Jahromi BN, Sarvari J. The prevalence of bacteriospermia in infertile men and association with semen quality in southwestern Iran. Infect Disord Drug Targets 2020; 20:198–202. 30474539 10.2174/1871526519666181123182116

[37] Hassan JS, Salah RF, Gaidan AM. The role of chlamydial infection in male infertility. Ind J Pub Health Res Dev 2019; 10:710–5.

[38] Moosavian M, Ghadiri A, Amirzadeh S, et al Investigating Chlamydia trachomatis and genital Mycoplasma prevalence and apoptosis markers in infertile and fertile couples. Jundishapur J Microbiol 2019; 12:e84954.

[39] Ahmadi MH, Mirsalehian A, Sadighi Gilani MA, Bahador A, Afraz K. Association of asymptomatic Chlamydia trachomatis infection with male infertility and the effect of antibiotic therapy in improvement of semen quality in infected infertile men. Andrologia 2018. 10.1111/and.12944 29292525

[40] Ali MH, Al-Kazaz AKA. Molecular detection of Chlamydia trachomatis infection among males with abnormal semen. Iraqi J Sci 2018; 59(4B):2005–11.

[41] Liu J, Wang Q, Ji X, et al Prevalence of Ureaplasma urealyticum, Mycoplasma hominis, Chlamydia trachomatis infections, and semen quality in infertile and fertile men in China. Urology 2014; 83:795–9. 24411218 10.1016/j.urology.2013.11.009

[42] Abusarah EA, Awwad ZM, Charvalos E, Shehabi AA. Molecular detection of potential sexually transmitted pathogens in semen and urine specimens of infertile and fertile males. Diagn Microbiol Infect Dis 2013; 77:283–6. 24079950 10.1016/j.diagmicrobio.2013.05.018

[43] Noruziyan Z, Roghanian R, Hosseinzadeh S, Golbang N, Nasr Esfahani MH. Possible role of Chlamydia trachomatis in the male partner of infertile couples. Comp Clin Pathol 2013; 22:421–4.

[44] Osazuwa F, Aiguobarueghian OI, Alekwe L, Imade PE, Ibadin KO, Aberare LO. The prevalence of Chlamydia trachomatis infection among infertile males and its association with abnormal semen characteristics in Delta State, Nigeria. Tanzan J Health Res 2013; 15:88–92. 26591714 10.4314/thrb.v15i2.3

[45] Al-Sweih NA, Al-Fadli AH, Omu AE, et al Prevalence of Chlamydia trachomatis, Mycoplasma hominis, Mycoplasma genitalium, and Ureaplasma urealyticum infections and seminal quality in infertile and fertile men in Kuwait. J Androl 2012; 33:1323–9. 22052774 10.2164/jandrol.111.013821

[46] Günyeli I, Abike F, Dünder I, et al Chlamydia, Mycoplasma and Ureaplasma infections in infertile couples and effects of these infections on fertility. Arch Gynecol Obstet 2011; 283:379–85. 20978774 10.1007/s00404-010-1726-4

[47] Çalışkan T, Koc¸ak _I, Kırdar S, et al Human papillomavirus and Chlamydia trachomatis in semen samples of asymptomatic fertile and infertile men: prevalence and relation between semen parameters and IL-18 levels. Turk J Urol 2010; 36:143–8.

[48] El Feky MA, Hassan EA, El Din AM, et al Chlamydia trachomatis: methods of identification and impact on semen quality. Egypt J Immunol 2009; 16:49–59. 20726322

[49] Ouzounova-Raykova V, Ouzounova I, Mitov I. Chlamydia trachomatis infection as a problem among male partners of infertile couples. Andrologia 2009; 41:14–9. 19143724 10.1111/j.1439-0272.2008.00881.x

[50] Liu DB, Zhu PY. [Detection of IgG and IgM antibodies against Chlamydia trachomatis in semen of asymptomatic infertile patients]. Zhonghua Nan Ke Xue 2003; 9:197–9. 12861834

[51] World Health Organization (WHO) . Infertility prevalence estimates, 1990–2021. Geneva: World Health Organization; 2023. iris.who.int/bitstream/handle/10665/366700/9789240068315-eng.pdf?sequence=1.

