# Supplementary material for: The Impact of Chlamydia trachomatis on Male Infertility: A Systematic Review and Meta-Analysis
Source: Open Forum Infect Dis. 2025 Dec 23;13(1):ofaf782. doi: 10.1093/ofid/ofaf782 (PMC12757865; doi:10.1093/ofid/ofaf782)
Supplement: ofaf782_Supplementary_Data [file ofaf782_supplementary_data.docx]

**Supplementary Table 1. Database-specific search strategies for identifying literature on *Chlamydia trachomatis* and male infertility in PubMed/MEDLINE, Embase, Scopus, and Cochrane Library.**

| **Database** | **Search string** |
| --- | --- |
| PubMed/MEDLINE | ("chlamydia"[MeSH Terms] OR "chlamydia"[All Fields] OR "chlamydiae"[All Fields] OR "chlamydias"[All Fields] OR ("chlamydia"[MeSH Terms] OR "chlamydia"[All Fields] OR "chlamydial"[All Fields]) OR "chlamydia*"[All Fields]) AND ("male"[MeSH Terms] OR "male"[All Fields] OR ("male"[MeSH Terms] OR "male"[All Fields] OR "males"[All Fields] OR "male s"[All Fields] OR "maleness"[All Fields]) OR "male*"[All Fields] OR ("men"[MeSH Terms] OR "men"[All Fields] OR "man"[All Fields]) OR ("men"[MeSH Terms] OR "men"[All Fields]) OR ("husband s"[All Fields] OR "spouses"[MeSH Terms] OR "spouses"[All Fields] OR "husband"[All Fields] OR "husbands"[All Fields]) OR ("husband s"[All Fields] OR "spouses"[MeSH Terms] OR "spouses"[All Fields] OR "husband"[All Fields] OR "husbands"[All Fields]) OR "husband*"[All Fields]) AND ("sperm s"[All Fields] OR "spermatozoa"[MeSH Terms] OR "spermatozoa"[All Fields] OR "sperm"[All Fields] OR "sperms"[All Fields] OR "sperm*"[All Fields] OR ("semen"[MeSH Terms] OR "semen"[All Fields] OR "semen s"[All Fields] OR "semens"[All Fields]) OR "seminal"[All Fields] OR "hypospermia"[All Fields] OR "hypospermic"[All Fields] OR "hyposperm*"[All Fields] OR ("oligospermia"[MeSH Terms] OR "oligospermia"[All Fields]) OR ("oligospermic"[All Fields] OR "oligospermics"[All Fields]) OR "oligosperm*"[All Fields] OR ("oligospermia"[MeSH Terms] OR "oligospermia"[All Fields] OR "oligozoospermia"[All Fields]) OR ("oligozoospermic"[All Fields] OR "oligozoospermics"[All Fields]) OR "oligozoospem*"[All Fields] OR "pyospermia"[All Fields] OR "pyospermic"[All Fields] OR "pyosperm*"[All Fields] OR "pyosemia"[All Fields] OR ("azoospermia"[MeSH Terms] OR "azoospermia"[All Fields] OR "azoospermias"[All Fields]) OR ("azoospermic"[All Fields] OR "azoospermics"[All Fields]) OR "azoosperm*"[All Fields] OR "leukocytospermia"[All Fields] OR "leukocytospermic"[All Fields] OR "leukocytosperm*"[All Fields] OR "normospermia"[All Fields] OR ("normospermic"[All Fields] OR "normospermics"[All Fields]) OR "normosperm*"[All Fields] OR "normozoospermia"[All Fields] OR ("normozoospermic"[All Fields] OR "normozoospermics"[All Fields]) OR "normozoosperm*"[All Fields] OR ("hemospermia"[MeSH Terms] OR "hemospermia"[All Fields] OR "haematospermia"[All Fields] OR "hematospermia"[All Fields]) OR "hematospermic"[All Fields] OR "hematosperm*"[All Fields] OR ("hemospermia"[MeSH Terms] OR "hemospermia"[All Fields] OR "haemospermia"[All Fields]) OR "haemosperm*"[All Fields] OR ("asthenozoospermia"[MeSH Terms] OR "asthenozoospermia"[All Fields]) OR ("asthenozoosperm"[All Fields] OR "asthenozoospermic"[All Fields]) OR "asthenozoosperm*"[All Fields] OR ("oligospermia"[MeSH Terms] OR "oligospermia"[All Fields] OR "oligoasthenoteratozoospermia"[All Fields]) OR "oligoasthenoteratozoospermic"[All Fields] OR "oligoasthenoteratozoosperm*"[All Fields] OR ("teratozoospermia"[MeSH Terms] OR "teratozoospermia"[All Fields]) OR "teratozoospermic"[All Fields] OR "teratozoosperm*"[All Fields] OR "oligoteratozoospermia"[All Fields] OR "oligoteratozoospermic"[All Fields] OR "oligoteratozoosperm*"[All Fields] OR "oligoasthenozoospermia"[All Fields] OR "oligoasthenozoospermic"[All Fields] OR "oligoasthenozoosperm*"[All Fields] OR ("oligospermia"[MeSH Terms] OR "oligospermia"[All Fields] OR "cryptozoospermia"[All Fields]) OR "cryptozoospermic"[All Fields] OR "cryptozoosperm*"[All Fields] OR ("fertiles"[All Fields] OR "fertility"[MeSH Terms] OR "fertility"[All Fields] OR "fertile"[All Fields] OR "fertilities"[All Fields]) OR ("fertiles"[All Fields] OR "fertility"[MeSH Terms] OR "fertility"[All Fields] OR "fertile"[All Fields] OR "fertilities"[All Fields]) OR "fertil*"[All Fields] OR ("infertiles"[All Fields] OR "infertilities"[All Fields] OR "infertility"[MeSH Terms] OR "infertility"[All Fields] OR "infertile"[All Fields] OR "infertility s"[All Fields]) OR ("infertiles"[All Fields] OR "infertilities"[All Fields] OR "infertility"[MeSH Terms] OR "infertility"[All Fields] OR "infertile"[All Fields] OR "infertility s"[All Fields]) OR "infertil*"[All Fields] OR ("infertility"[MeSH Terms] OR "infertility"[All Fields] OR "subfertility"[All Fields] OR "subfertile"[All Fields]) OR ("infertility"[MeSH Terms] OR "infertility"[All Fields] OR "subfertility"[All Fields] OR "subfertile"[All Fields]) OR "subfertil*"[All Fields]) |
| Embase | ('chlamydia'/exp OR chlamydia OR chlamydial OR chlamydia*) AND ('male'/exp OR male OR 'males'/exp OR males OR male* OR 'man'/exp OR man OR 'men'/exp OR men OR 'husband'/exp OR husband OR husbands OR husband*) AND ('sperm'/exp OR sperm OR sperm* OR 'semen'/exp OR semen OR seminal OR 'hypospermia'/exp OR hypospermia OR hypospermic OR hyposperm* OR 'oligospermia'/exp OR oligospermia OR oligospermic OR oligosperm* OR 'oligozoospermia'/exp OR oligozoospermia OR oligozoospermic OR oligozoospem* OR 'pyospermia'/exp OR pyospermia OR pyospermic OR pyosperm* OR pyosemia OR 'azoospermia'/exp OR azoospermia OR azoospermic OR azoosperm* OR 'leukocytospermia'/exp OR leukocytospermia OR leukocytospermic OR leukocytosperm* OR 'normospermia'/exp OR normospermia OR normospermic OR normosperm* OR 'normozoospermia'/exp OR normozoospermia OR normozoospermic OR normozoosperm* OR 'hematospermia'/exp OR hematospermia OR hematospermic OR hematosperm* OR 'haemospermia'/exp OR haemospermia OR haemosperm* OR 'asthenozoospermia'/exp OR asthenozoospermia OR asthenozoospermic OR asthenozoosperm* OR 'oligoasthenoteratozoospermia'/exp OR oligoasthenoteratozoospermia OR oligoasthenoteratozoospermic OR oligoasthenoteratozoosperm* OR 'teratozoospermia'/exp OR teratozoospermia OR teratozoospermic OR teratozoosperm* OR 'oligoteratozoospermia'/exp OR oligoteratozoospermia OR oligoteratozoospermic OR oligoteratozoosperm* OR 'oligoasthenozoospermia'/exp OR oligoasthenozoospermia OR oligoasthenozoospermic OR oligoasthenozoosperm* OR 'cryptozoospermia'/exp OR cryptozoospermia OR cryptozoospermic OR cryptozoosperm* OR fertile OR 'fertility'/exp OR fertility OR fertil* OR infertile OR 'infertility'/exp OR infertility OR infertil* OR subfertile OR 'subfertility'/exp OR subfertility OR subfertil*) |
| Scopus | TITLE-ABS-KEY ( ( chlamydia OR chlamydial OR chlamydia* ) AND ( male OR males OR male* OR man OR men OR husband OR husbands OR husband* ) AND ( sperm OR sperm* OR semen OR seminal OR hypospermia OR hypospermic OR hyposperm* OR oligospermia OR oligospermic OR oligosperm* OR oligozoospermia OR oligozoospermic OR oligozoospem* OR pyospermia OR pyospermic OR pyosperm* OR pyosemia OR azoospermia OR azoospermic OR azoosperm* OR leukocytospermia OR leukocytospermic OR leukocytosperm* OR normospermia OR normospermic OR normosperm* OR normozoospermia OR normozoospermic OR normozoosperm* OR hematospermia OR hematospermic OR hematosperm* OR haemospermia OR haemosperm* OR asthenozoospermia OR asthenozoospermic OR asthenozoosperm* OR oligoasthenoteratozoospermia OR oligoasthenoteratozoospermic OR oligoasthenoteratozoosperm* OR teratozoospermia OR teratozoospermic OR teratozoosperm* OR oligoteratozoospermia OR oligoteratozoospermic OR oligoteratozoosperm* OR oligoasthenozoospermia OR oligoasthenozoospermic OR oligoasthenozoosperm* OR cryptozoospermia OR cryptozoospermic OR cryptozoosperm* OR fertile OR fertility OR fertil* OR infertile OR infertility OR infertil* OR subfertile OR subfertility OR subfertil* ) ) |
| The Cochrane Library | (chlamydia OR chlamydial OR chlamydia*) AND (male OR males OR male* OR man OR men OR husband OR husbands OR husband*) AND (sperm OR sperm* OR semen OR seminal OR hypospermia OR hypospermic OR hyposperm* OR oligospermia OR oligospermic OR oligosperm* OR oligozoospermia OR oligozoospermic OR oligozoospem* OR pyospermia OR pyospermic OR pyosperm* OR pyosemia OR azoospermia OR azoospermic OR azoosperm* OR leukocytospermia OR leukocytospermic OR leukocytosperm* OR normospermia OR normospermic OR normosperm* OR normozoospermia OR normozoospermic OR normozoosperm* OR hematospermia OR hematospermic OR hematosperm* OR haemospermia OR haemosperm* OR asthenozoospermia OR asthenozoospermic OR asthenozoosperm* OR oligoasthenoteratozoospermia OR oligoasthenoteratozoospermic OR oligoasthenoteratozoosperm* OR teratozoospermia OR teratozoospermic OR teratozoosperm* OR oligoteratozoospermia OR oligoteratozoospermic OR oligoteratozoosperm* OR oligoasthenozoospermia OR oligoasthenozoospermic OR oligoasthenozoosperm* OR cryptozoospermia OR cryptozoospermic OR cryptozoosperm* OR fertile OR fertility OR fertil* OR infertile OR infertility OR infertil* OR subfertile OR subfertility OR subfertil*) in Title Abstract Keyword |

**Supplementary Table 2. Characteristics of the studies included in the present systematic literature review and meta-analysis on the association between *Chlamydia trachomatis* and male infertility.**

| **Study** | **Study publication year** | **Study year** | **Country** | **Infertility case definition** | **Sperm sample collection** | **Exclusion criteria** | **Control definition** | **Sample size** | **A priori sample power analysis** | **Age** | **Age matching** | **Cases** | **Controls** | **Cases with CT** | **Controls with CT** | **PCR** | **ELISA** |
| --- | --- | --- | --- | --- | --- | --- | --- | --- | --- | --- | --- | --- | --- | --- | --- | --- | --- |
| Naik et al., 2022 | 2022 | June 2018 - September 2019 | India (Odisha) | Not specified | Not specified | Patients with already diagnosed sperm dysfunction, primary and secondary testicular failure, STD (Mycoplasma and Gonococci) | Of reproductive age and with at least one biological child, without any medical issue | 139 | No | 35 years (median; IQR 29 - 59 years) | NA | 79 | 60 | 1 | 0 | PCR for urine and seminal fluid | ELISA for IgG, IgA, and IgM antibodies in serum samples |
|  |  |  |  |  |  |  |  |  |  |  |  | 79 | 74 | 0 | 0 | Urine |  |
|  |  |  |  |  |  |  |  |  |  |  |  | 79 | 60 | 1 | 0 | Seminal fluid |  |
|  |  |  |  |  |  |  |  |  |  |  |  | 72 | 24 | 9 | 0 |  | IgG |
|  |  |  |  |  |  |  |  |  |  |  |  | 75 | 16 | 3 | 0 |  | IgM |
|  |  |  |  |  |  |  |  |  |  |  |  | 71 | 13 | 20 | 0 |  | IgA |
| Ahmadi et al., 2023 | 2023 | August 2016 - March 2017 | Iran (Khuzestan Province) | Clinician confirmed (andrologist) | Sexual abstinence of 2-3 days before the testing | Use of antibiotics for one week before the sampling | No history of infertility, with established fertility (at least one biological child), without defects in sperm parameters and no use of antibiotics | 100 | Yes | Entire population 20–40 (31±4.1); 33 years (range 25-40 years) in the infertility group, 31 years (range 19–40 years) in the fertility group | Yes | 50 | 50 | 5 | 0 | PCR for seminal fluid |  |
| Li et al., 2023 | 2023 | October 2014 - January 2023 | China (Shandong Province) | Clinician and lab confirmed | Sexual abstinence of 2-7 days before the testing | Female-related factors, organic reproductive system diseases, congenital gonadal dysplasia, varicocele, and other diseases affecting semen quality, including azoospermia | Healthy, without any factors affecting semen quality, such as female factors, congenital gonadal dysgenesis, varicocele, or azoospermia | 883 | No | 33.0 ± 5.4 years (range 22-48 years) in the infertility group, 34.6 ± 5.1 years (19-52 years) in the control group | Yes | 393 | 490 | 24 | 3 | Fluorescent quantitative PCR and NAAT fluorescent kit for uro-genital swabs (urethral secretions or genital tract scrapings) |  |
| Zeng et al., 2024 | 2024 | December 2021 - April 2023 | China (Putian, Fujian Province) | Clinician confirmed | Not specified | Not specified | Clinical ascertainment | 611 | No | Entire population 16–61 (31.36±4.93) years | NA | 456 | 155 | 10 | 9 | Real-time fluorescence PCR for genital secretions and fluids |  |
| Dehghan et al., 2022 | 2022 | 2022 | Iran (Tehran) | WHO-based | Sexual abstinence of 2-7 days before the testing | Exposure to alcohol, smoking, physical and chemical factors affecting fertility, past steriliization, ration, or chemotherapy treatment, varicocele testicular tumors, genital abnormalities, sperm autoantibodies, symptoms of genitourinary diseases, antibiotics in the last month, and sexually transmitted infections | With normal semen parameters and with wives who had pregnancies without using auxiliary procedures in the past | 130 | No | 35.2 ± 6.8 years (24-56 years) | NA | 65 | 65 | 6 | 2 | Real-time PCR for seminal fluid |  |
| López-Hurtado et al., 2021 | 2021 | January 2018 - December 2018 | Mexico (Mexico City) | Not specified | Not specified | Not specified | Not specified | 659 | No | 32.6 years (range 20-67 years) | NA | 287 | 372 | 43 | 6 | Real-time nucleic acid amplification test (qPCR) and PCR-RFLP for genotyping from urine samples |  |
| EzzEl-Din et al., 2021 | 2021 | 2021 | Egypt (Assiut) | Lab confirmed | Sexual abstinence of 3-5 days before the testing | Varicocele, azoospermia, smoking, and use of drugs affecting spermatogenesis for at least the last three months prior to study | No alterations in semen parameters and no pyospermia | 275 | No | 35.30 ± 5.90 years (33) in the infertility group; 36.20± 5.74 years (35.5) in the control group | Yes | 250 | 25 | 60 | 0 | PCR for CT plasmid DNA detection in the semen sediment | For CT IgA detection in the seminal plasma |
| Pebdeni et al., 2022 | 2022 | July 2019 - December 2019 | Iran (Kerman) | According to the WHO (failure to conceive after at least 12 months of unprotected sexual intercourse) | Sexual abstinence of 2-7 days before the testing | Female factor subfertility, hormonal abnormalities, and reproductive system abnormalities (varicocele, hydrocele, undescended testis, or inguinal hernia), clinical signs of genitourinary tract infections; antibiotics in the last 2 weeks | Normal semen parameters; wives had non-assisted pregnancies | 200 | Yes | 35.33 ± 7.38 years (range 21-60 years) in the infertile group, 34.29 ± 7.3 years (range 17-60 years) in the fertile group | Yes | 100 | 100 | 9 | 0 | PCR for seminal fluid |  |
| El-Din et al., 2021 | 2021 | January 2018 - January 2020 | Egypt (Sohag) | WHO-based, clinician confirmed (andrologist) | Sexual abstinence of 3-5 days before the testing | Urogenital tract infections, endocrine disorders, chromosomal anomalies, reproductive system abnormalities (varicoceles, hydroceles, undescended testis or testicular tumours), antibiotic use over the last two weeks, and azoospermia; smoking, diabetes, hypertension, and obesity were NOT a reason for exclusion and differed significantly between cases and controls | Normal semen~~t~~ parameters and who achieved natural pregnancies without medical assistance | 265 | No | 38 ± 2 years (infertile men), 29 ± 1 years (fertile men) | No | 200 | 65 | 15 | 0 | PCR for seminal fluid | ELISA for anti-Chlamydia IgA antibodies in seminal plasma |
|  |  |  |  |  |  |  |  |  |  |  |  | 200 | 65 | 15 | 0 | PCR |  |
|  |  |  |  |  |  |  |  |  |  |  |  | 200 | 65 | 28 | 6 |  | IgA |
| Paira et al., 2021 | 2021 | January 2015 - November 2019 | Argentina (Cordoba) | WHO-based, clinician confirmed (uro-andrologist) | Sexual abstinence of 2-7 days before the testing | Symptoms of genital tract infections, antibiotics in the last 3 weeks | Healthy, asymptomatic, no complaints of infertility, no antibiotics in the last 3 weeks | 3950 | No | 18-60 years | NA | 3610 | 340 | 208 | 6 | PCR for seminal fluid |  |
| Hassan et al., 2019 | 2019 | July 2016 - February 2017 | Iraq (Baghdad) | WHO-based, clinician confirmed (a sterility and urinary tract specialist) | Not specified | Known disturbance in hormonal levels, anatomical problems, such as varicocele and cryptorchidism, karyotyping abnormalities, a previous or ongoing treatment for fertility disorders and the presence of sperm defects of supposed genetic origin | Without genito-urinary tract anatomical deformities or infection | 200 | No | 28.92 ± 5.9 years (infertile men), 27.73 ± 3.9 years (fertile men) | Yes | 100 | 100 | 17 | 1 | PCR for seminal fluid |  |
| López-Hurtado et al., 2020 | 2020 | April 2018-September 2018 | Mexico | Lab and imaging confirmed | Not specified | Use of antibiotics in the past 30 days, a known immunosuppressive disease, and signs of an emotional or mental health crisis or some type of cancer treatment | Not specified | 668 | No | 20-52 years | NA | 269 | 399 | 25 | 33 | Real-time PCR from urine samples |  |
| Motamedifar et al., 2020 | 2020 | 2015 | Southwestern Iran | Not specified | Sexual abstinence of 3 days before the testing | Use of antibiotics in the week before the testing; | Not specified | 350 | No | 36±7.0 years (range 22-61 years) in the infertility group, 36±6.9 years (range 24-62 yeas) in the fertility group | Yes | 200 | 150 | 25 | 2 | PCR for seminal fluid |  |
| Moosavian et al., 2019 | 2019 | August 2016 - March 2017 | Iran (Ahvaz) | According to the WHO (failure to conceive after at leastone year of unprotected intercourse), clinically confirmed by an andrologist | Sexual abstention for at least 48 hours before the tests (4-5 days) | Antibiotic therapy for one week before sampling | Lack of infertility and antibiotic use | 100 | Yes | 31.4 years (range 20-40 years) | NA | 50 | 50 | 5 | 0 | PCR for seminal fluid |  |
| Ali et al., 2018 | 2018 | 2018 | Iraq (Baghdad) | Difficulty to conceive, no children, abnormal seminal parameters | Not specified | Not specified | At least one child and normal seminal fluid | 76 | No | 32.28 ± 6.88 years (range 20-52 years) in the infertile group, 34.07 ± 6.52 years (range 21-45 years) in the fertile group | Yes | 63 | 13 | 11 | 0 | Real-time PCR for seminal fluid |  |
| Ahmadi et al., 2018 | 2018 | 2018 | Iran (Tehran) | Lab confirmed (abnormal semen parameters) | Sexual abstinence of 3-7 days | Symptoms of urogenital tract infections, endocrine disorders, chromosomal anomalies, reproductive system abnormalities (varicocele, hydrocele or undescended testis), testicular tumours, systemic diseases, sperm autoantibodies, antibiotic use within the previous week, azoospermia, heavy use of alcohol, heavy smoking or continuous exposure to chemical or physical agents with known adverse reproductive effects (e.g., benzene and radiation) | Normal semen parameters; wives had non-assisted pregnancies | 330 | No | 34.3 ± 0.4 years (range 24-59 years) in infertile men, 33.6 ± 0.4 years (24-49 years) in fertile men | Yes | 165 | 165 | 7 | 1 | Real-time PCR for seminal fluid |  |
| Al-Sweih et al., 2012 | 2012 | October 2008 - November 2009 | Kuwait | WHO-based (failure to impregnate their wives after at least 12 months of unprotected sexual intercourse) and lab confirmed (abnormal semen parameters) | Sexual abstinence of 5 days | Symptoms of STDs or any genital tract infections and use of antibiotics in the last 2 weeks | Men who had consummated their marriages and whose wives had non-assisted pregnancies | 315 | No | NA | NA | 127 | 188 | 4 | 5 | PCR for seminal fluid |  |
| Osazuwa et al., 2013 | 2013 | November 2011 - November 2012 | Nigeria (Delta State) | Lab confirmed (abnormal semen parameters) | Sexual abstinence of 3-5 days | No symptoms of genital infection | No complaints of infertility | 255 | No | 20-29 years: 13 (43.3%) 30-39 years: 21 (22.1%) 40-49 years: 5 (7.9%) ≥50 years: 1 (3.7%) | NA | 215 | 40 | 42 | 2 |  | Dot rapid Assay Kit flow through Ct cassette and enzyme immunoassay from blood (IgG) |
|  |  |  |  |  |  |  |  |  |  |  |  | 215 | 40 | 42 | 2 |  | Dot rapid assay kit flow |
|  |  |  |  |  |  |  |  |  |  |  |  | 215 | 40 | 42 | 2 |  | IgG Enzyme immunoassay |
| Noruziyan et al., 2013 | 2013 | 2011 - 2012 | Iran (Isfahan) | WHO-based (failure to conceive with their partner after 1 year of unprotected intercourse) | Not specified | Symptoms of genital infections, history of treatment by chemotherapy (or radiotherapy to the groin), vasostomy or orchidectomy | Not specified | 186 | No | 32.8 ± 6.3 years (infertile men), 36.7 ± 6.5 years (fertile men) | No | 93 | 93 | 18 | 7 | PCR for seminal fluid | ELISA for IgA and IgG antibodies in the blood |
|  |  |  |  |  |  |  |  |  |  |  |  | 93 | 93 | 18 | 7 | PCR |  |
|  |  |  |  |  |  |  |  |  |  |  |  | 93 | 93 | 4 | 3 |  | IgG |
|  |  |  |  |  |  |  |  |  |  |  |  | 93 | 93 | 2 | 1 |  | IgA |
| Abusarah et al., 2013 | 2013 | May 2011 - October 2011 | Jordan (Amman and Zarka regions) | Lab confirmed (history of infertility, abnormal semen parameters) | Not specified | Clinical signs of genitourinary tract infections, use of antibiotics in the past 2 weeks | Normal semen parameter and/or with wives who had non-assisted pregnancies in the past | 163 | No | 33 ± 8.07 years (range 20-58 years) in the infertile group, 32 ± 6.74 years (range 20-48 years) in the fertile group | Yes | 93 | 70 | 4 | 1 | PCR |  |
|  |  |  |  |  |  |  |  |  |  |  |  | 93 | 70 | 4 | 1 | Semen |  |
|  |  |  |  |  |  |  |  |  |  |  |  | 81 | 61 | 4 | 1 | Urine |  |
| Günyeli et al., 2011 | 2011 | 2010 | Turkey (Ankara) | Not specified | Sexual abstinence of 3 days | Not specified | Not specified | 106 | No | 30.43 ± 5.58 years for infertile men, 37.66 ± 7.02 for fertile men | No | 53 | 53 | 2 | 1 |  | ELISA for IgM antibodies in blood, immunochromatographic method for CT antigen detection in urethral swabs, and cell culture (the ‘‘Bouillon uree-arginine Lyo’’ medium for urethral swabs) |
|  |  |  |  |  |  |  |  |  |  |  |  | 53 | 53 | 2 | 1 |  | IgM |
| Çalışkan et al., 2010 | 2010 | 2006 | Turkey (Aydın) | Lab confirmed (abnormal semen parameters) | Sexual abstinence of 3-4 days | Genital warts and symptoms of genital infections | Not specified | 175 | No | Entire population 19-47 years; 32 years (IQR 18-50 years) in the infertility group; 32.0 years (IQR 23-46.0 years) in the fertility group | Yes | 144 | 31 | 12 | 3 | PCR for seminal fluid |  |
| El Feky et al., 2009 | 2009 | June 2007 - May 2008 | Egypt (Assiut) | Lab confirmed | Sexual abstinence of 3-5 days | Azoospermia, varicocele and drugs affecting spermatogenesis for at least the last three months prior to study, exposure to occupational agents known to affect spermatogenesis; antibiotics in the last week | Healthy | 100 | No | 30.8 ± 5.93 years (range 24 to 49 years) in the infertile group, 33.08 ± 7.72 years in the fertile group | Yes | 75 | 25 | 23 | 0 | PCR for seminal fluid | ELISA for IgA antibodies in seminal plasma and Flowcytometry for seminal fluid |
|  |  |  |  |  |  |  |  |  |  |  |  | 75 | 25 | 23 | 0 | PCR |  |
|  |  |  |  |  |  |  |  |  |  |  |  | 75 | 25 | 35 | 3 |  | Flowcytometry |
|  |  |  |  |  |  |  |  |  |  |  |  | 75 | 25 | 25 | 2 |  | IgA |
| Ouzounova-Raykova et al., 2009 | 2009 | 2009 | Bulgaria (Sofia) | Clinician and lab confirmed | Sexual abstinence of 3-4 days (from 48 hours to 7 days) | STDs; fevers and infections; surgery of the reproductive tract; damage to the vas deferens; varicocele; use of anti-depressant anti-hypertensive medications; exposure of the testes to high temperatures; use of tobacco, marijuana, or alcohol; medical conditions (diabetes); genetic or hormonal problems; and testicular injury were assessed but were NOT causes for exclusion | Healthy | 100 | No | 31 years (range 23-39 years) in the infertile group, 30 years (range 24-35 years) in the fertile group | Yes | 60 | 40 | 5 | 1 | Two different PCR protocols and cell culture (McCoy) both from urethral swabs |  |
|  |  |  |  |  |  |  |  |  |  |  |  | 60 | 40 | 5 | 1 | PCR |  |
|  |  |  |  |  |  |  |  |  |  |  |  | 60 | 40 | 4 | 1 | Cell culture |  |
| Liu and Zhu, 2003 | 2003 | March 2002 - October 2002 | China | Clinician confirmed (inability to conceive despite regular unprotected intercourse) | Sexual abstinence of at least 24 hours | Symptoms of genital infection | Fertile males with confirmed natural conception and normal seminal parameters | 134 | No | 29.7±3.4 (24-37) years in the infertility group, 28.6±3.4 (25-35) years in the fertility group | Yes | 116 | 18 | 30 | 0 |  | ELISA for IgG, IgM antibodies in seminal plasma and immunochromatographic assay for CT LPS antigen detection from urethral swab and urine samples |
|  |  |  |  |  |  |  |  |  |  |  |  | 116 | 18 | 30 | 0 |  | Immuchromatography |
|  |  |  |  |  |  |  |  |  |  |  |  | 116 | 118 | 16 | 2 |  | IgG |
|  |  |  |  |  |  |  |  |  |  |  |  | 116 | 118 | 4 | 0 |  | IgM |
| Liu et al., 2014 | 2014 | January 2011 - June 2013 | China | WHO-based (failure to impregnate their wives after at least 12 months of unprotected sexual intercourse) | Sexual abstinence of 3-5 days before the testing | Reproductive system abnormalities, heavy use of alcohol, heavy smoking, and exposure to physical or chemical agents with known negative reproductive effects | With wives who had non-assisted pregnancies | 1236 |  | NA | NA | 621 | 615 | 16 | 14 | PCR for urehtral swabs |  |

**Supplemental Table 3. Quality assessment of the studies included in the present systematic review and meta-analysis based on the Joanna Briggs Institute Checklist for Case Control Studies.**

| **Study** | **Item 1** | **Item 2** | **Item 3** | **Item 4** | **Item 5** | **Item 6** | **Item 7** | **Item 8** | **Item 9** | **Item 10** | **Overall appraisal score** |
| --- | --- | --- | --- | --- | --- | --- | --- | --- | --- | --- | --- |
| Zeng et al., 2024^24^ | Unclear | No | Yes | Yes | Yes | No | No | Yes | No | No | 40% |
| Ahmadi et al., 2023^25^ | Yes | Unclear | Yes | Yes | Yes | No | No | Yes | No | No | 50% |
| Li et al., 2023^26^ | Yes | Unclear | Yes | Yes | Yes | No | No | Yes | No | No | 50% |
| Dehghan et al., 2022^27^ | Unclear | No | Yes | Yes | Yes | Yes | Yes | Yes | No | No | 60% |
| Haidari Pebdeni et al., 2022^28^ | Yes | Unclear | Yes | Yes | Yes | No | No | Yes | No | Unclear | 50% |
| Naik et al., 2022^29^ | Unclear | No | Yes | Yes | Yes | No | No | Unclear | No | No | 30% |
| El-Din et al., 2021^30^ | No | No | Yes | Yes | Yes | No | No | Yes | No | No | 40% |
| EzzEl-Din et al., 2021^31^ | Yes | Yes | Yes | Yes | Yes | No | No | Yes | No | No | 60% |
| López-Hurtado et al., 2021^32^ | Unclear | No | Yes | Yes | Yes | No | No | Unclear | No | No | 30% |
| Paira et al., 2021^33^ | Unclear | No | Yes | Yes | Yes | No | No | Yes | No | No | 40% |
| López-Hurtado et al., 2020^34^ | Unclear | No | Yes | Yes | Yes | No | No | Yes | No | No | 40% |
| Motamedifar et al., 2020^35^ | Yes | Unclear | Yes | Yes | Yes | No | No | Unclear | No | No | 40% |
| Hassan et al., 2019^36^ | Yes | Unclear | Yes | Yes | Yes | No | No | Yes | No | No | 50% |
| Moosavian et al., 2019^37^ | Unclear | No | Yes | Yes | Yes | No | No | Yes | No | No | 40% |
| Ahmadi et al., 2018^38^ | Yes | Unclear | Yes | Yes | Yes | Yes | Yes | Yes | No | No | 70% |
| Ali et al., 2018^39^ | Yes | Unclear | Yes | Yes | Yes | No | No | Yes | No | No | 50% |
| Liu et al., 2014^40^ | Unclear | No | Yes | Yes | Yes | Yes | Yes | Yes | No | No | 60% |
| Abusarah et al., 2013^41^ | Yes | Unclear | Yes | Yes | Yes | No | No | Unclear | No | No | 40% |
| Noruziyan et al., 2013^42^ | No | No | Yes | Yes | Yes | No | No | Yes | No | No | 40% |
| Osazuwa et al., 2013^43^ | Unclear | No | Yes | Yes | Yes | No | No | Yes | No | No | 40% |
| Al-Sweih et al., 2012^44^ | Unclear | No | Yes | Yes | Yes | No | No | Yes | No | No | 40% |
| Günyeli et al., 2011^45^ | No | No | Yes | Yes | Yes | No | No | Unclear | No | No | 30% |
| Çalışkan et al., 2010^46^ | Yes | Unclear | Yes | Yes | Yes | No | No | Yes | No | No | 50% |
| El Feky et al., 2009^47^ | Yes | Yes | Yes | Yes | Yes | No | No | Yes | No | No | 60% |
| Ouzounova-Raykova et al., 2009^48^ | Yes | Unclear | Yes | Yes | Yes | Yes | No | Yes | No | No | 60% |
| Liu and Zhu, 2003^49^ | Yes | Unclear | Yes | Yes | Yes | No | No | Yes | No | No | 50% |

**Item 1. Were the groups comparable other than the presence of disease in cases or the absence of disease in controls? Item 2. Were cases and controls matched appropriately? Item 3. Were the same criteria used for identification of cases and controls? Item 4. Was exposure measured in a standard, valid and reliable way? Item 5. Was exposure measured in the same way for cases and controls? Item 6. Were confounding factors identified? Item 7. Were strategies to deal with confounding factors stated? Item 8. Were outcomes assessed in a standard, valid and reliable way for cases and controls? Item 9. Was the exposure period of interest long enough to be meaningful? Item 10. Was appropriate statistical analysis used?**

**Supplemental Figure 1. Sensitivity analysis of effect sizes (ES) and 95% confidence intervals (CI) for the association between *Chlamydia trachomatis* infection and male infertility.**


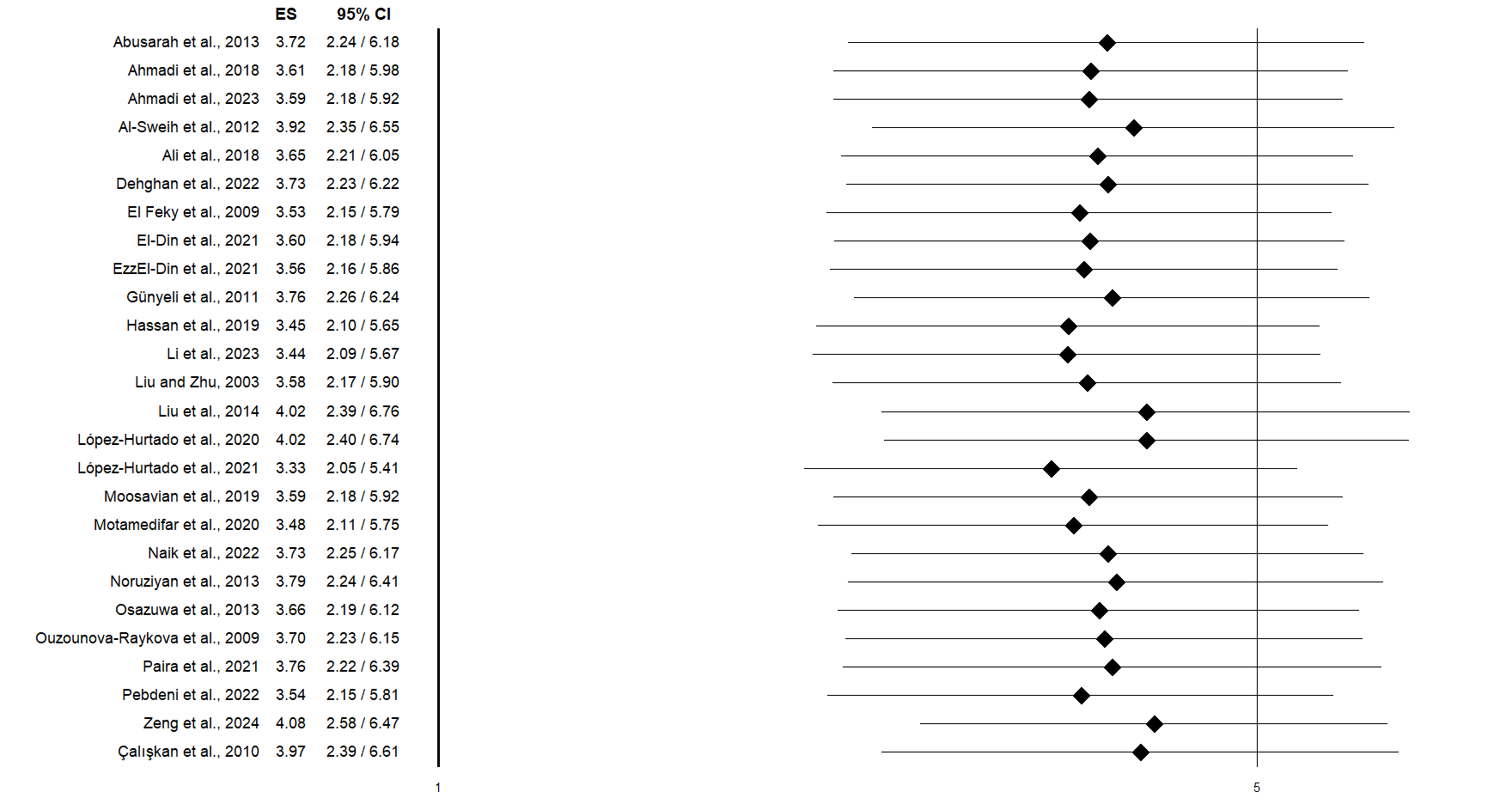


***This forest plot illustrates a leave-one-out sensitivity analysis, where each study is sequentially removed to assess its influence on the overall meta-analysis results. By systematically excluding individual studies, this approach evaluates the robustness and stability of the pooled effect size. The effect size (diamond) for each line represents the recalculated pooled estimate after removing the corresponding study. Minimal variation across the effect sizes suggests that no single study disproportionately impacts the overall findings, indicating a stable and robust meta-analysis.***

**Supplemental figure 2. Meta-regression of the association between publication year and effect size in studies on *Chlamydia trachomatis* and male infertility.**

**
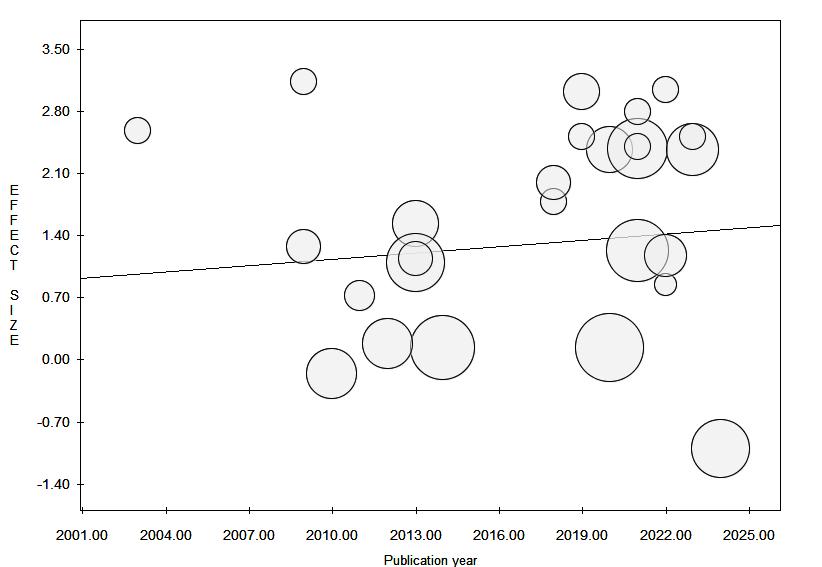
**

***Each circle represents a study, with the size proportional to the study's weight in the meta-analysis. The x-axis represents the publication year of the study, while the y-axis shows the corresponding effect size. The fitted regression line indicates the trend over time, assessing whether effect sizes have changed with more recent studies, showing no impact of publication year (p=0.600).***

**Supplemental Figure 3. Meta-regression of the association between age and effect size in studies on *Chlamydia trachomatis* and male infertility**


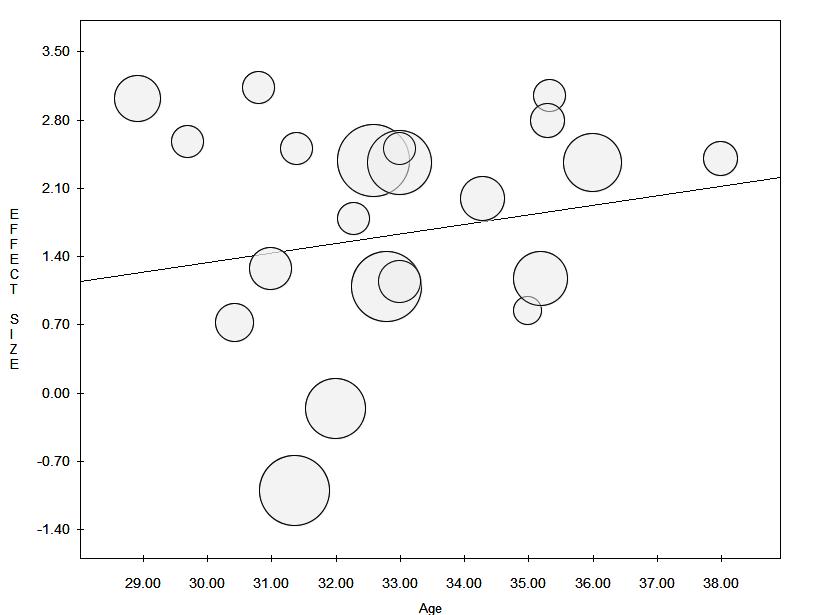


***Each circle represents a study, with the size proportional to the study's weight in the meta-analysis. The x-axis represents the mean age of participants, while the y-axis shows the corresponding effect size. The fitted regression line indicates the trend between age and effect size, showing no impact of age (p=0.451).***

**Supplemental figure 3. Meta-regression of the association between sample size and effect size in studies on *Chlamydia trachomatis* and male infertility.**


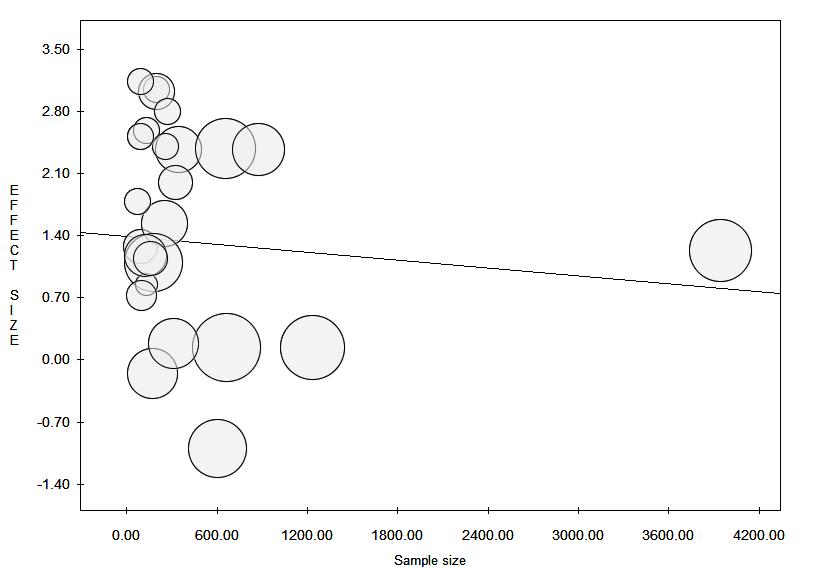


***Each circle represents a study, with the size proportional to the study's weight in the meta-analysis. The x-axis represents the total sample size of the study, while the y-axis shows the corresponding effect size. The fitted regression line examines whether larger studies report different effect sizes compared to smaller studies, helping assess potential small-study effects, and showing no impact of sample size (p=0.563).***

**Supplemental Figure 4. Subgroup analysis of effect sizes (ES) and 95% confidence intervals (CI) for the association between *Chlamydia trachomatis* and male infertility, stratified by country.**


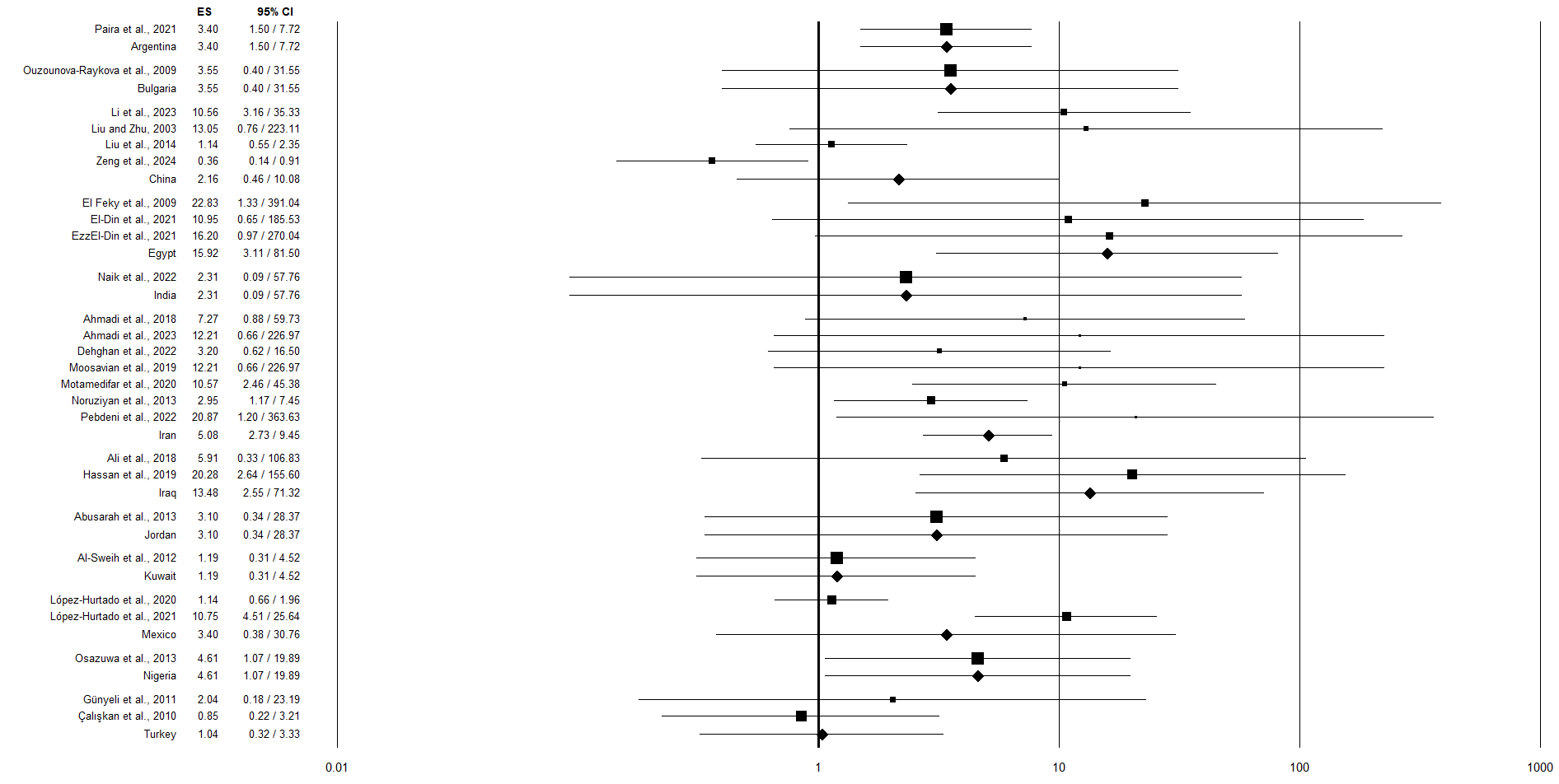


***This forest plot presents a subgroup analysis based on the country where each study was conducted. Each study’s ES and corresponding 95%CI are displayed, with pooled estimates shown for each country (diamonds). Larger squares indicate studies with greater weight in the analysis. This analysis assesses potential geographical variations in the association between Chlamydia trachomatis infection and male infertility, showing no impact of study country (p=0.211).***

**Supplemental Figure 5. Subgroup analysis of effect sizes (ES) and 95% confidence intervals (CI) for the association between *Chlamydia trachomatis* and male infertility, stratified by diagnostic method.**


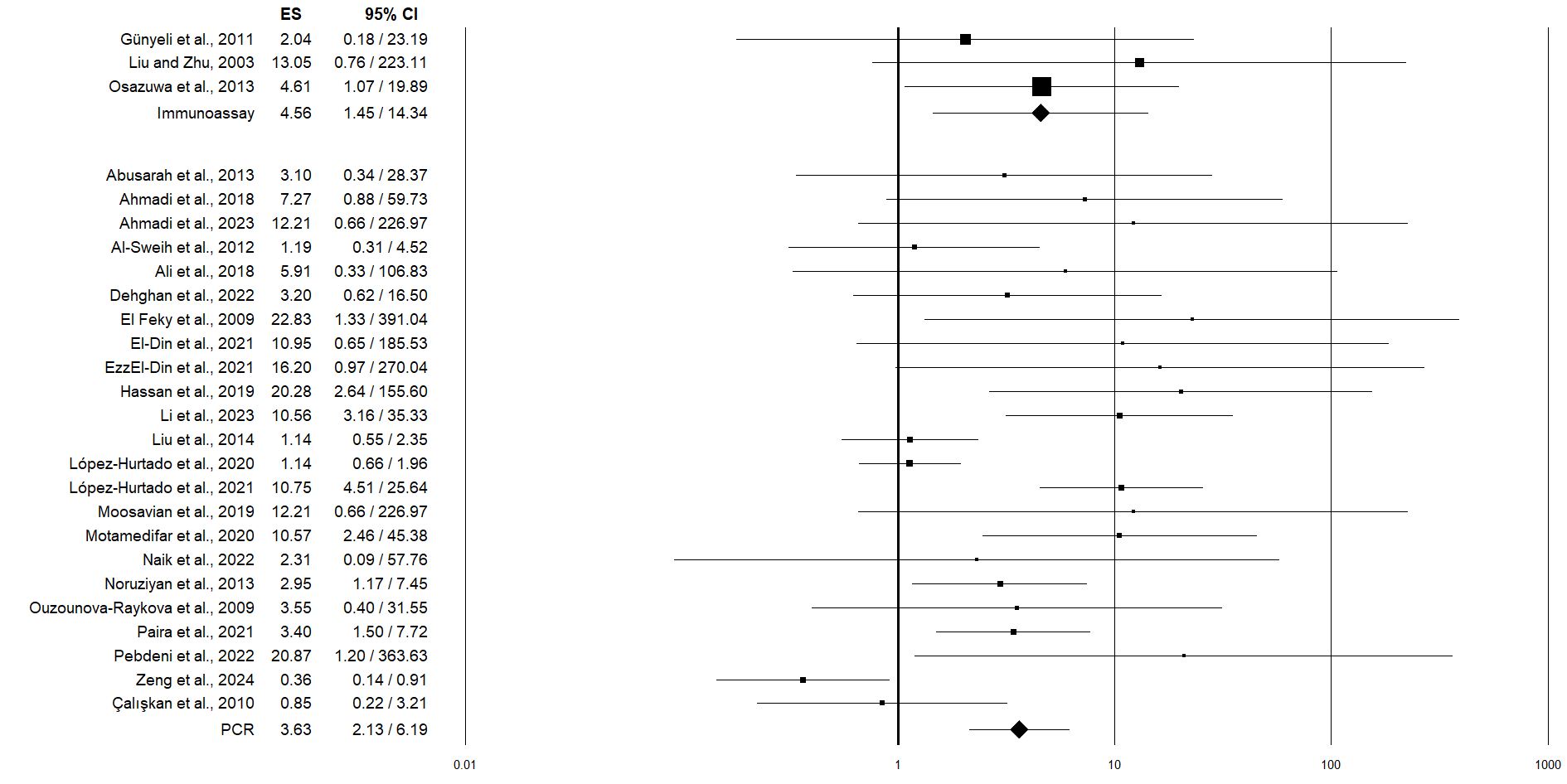


***This forest plot presents a subgroup analysis comparing studies based on the diagnostic method used to detect Chlamydia trachomatis (PCR vs. immunoassay). Each study’s ES) and corresponding 95%CI are displayed, with pooled estimates shown for each subgroup (diamonds). Larger squares indicate studies with greater weight in the analysis. The results do not highlight potential differences in ESs depending on the detection method (p=0.922).***

**Supplemental figure 6. Subgroup analysis of effect sizes (ES) and 95% confidence intervals (CI) for the association between *Chlamydia trachomatis* and male infertility, stratified by clinician-confirmed infertility.**


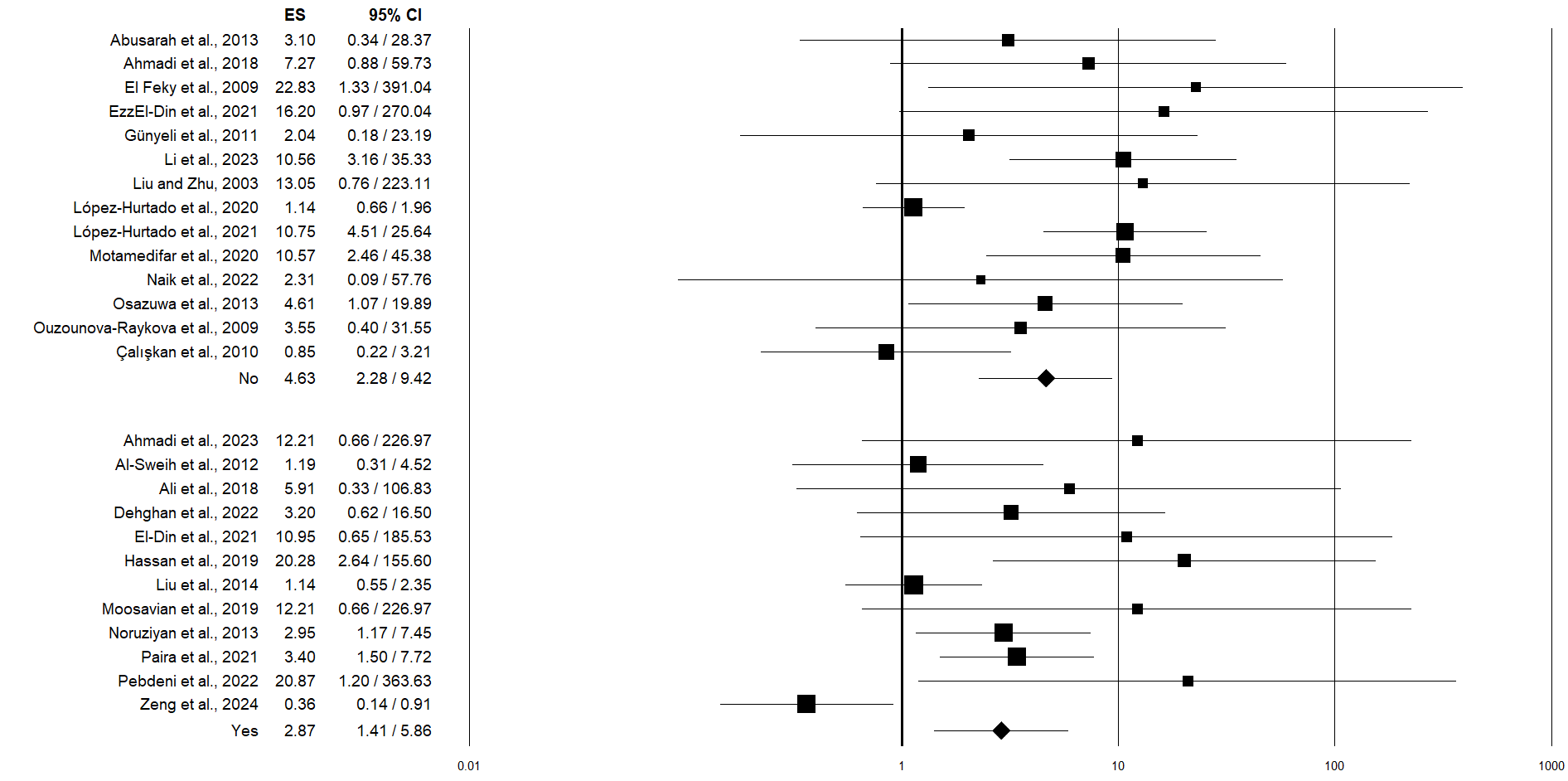


***This forest plot presents a subgroup analysis based on whether infertility was confirmed by a clinician. Studies are divided into those where infertility was clinically diagnosed (“Yes”) and those where it was not explicitly confirmed by a clinician (“No”). Each study’s ES and corresponding 95%CI are displayed, with pooled estimates shown for each subgroup (diamonds). Larger squares indicate studies with greater weight in the analysis. The results demonstrate that clinician confirmation does not significantly influence the observed association (p=0.351), suggesting consistency across both subgroups.***

**Supplemental Figure 7. Subgroup analysis of effect sizes (ES) and 95% confidence intervals (CI) for the association between *Chlamydia trachomatis* and male infertility, stratified by age-matching status.**


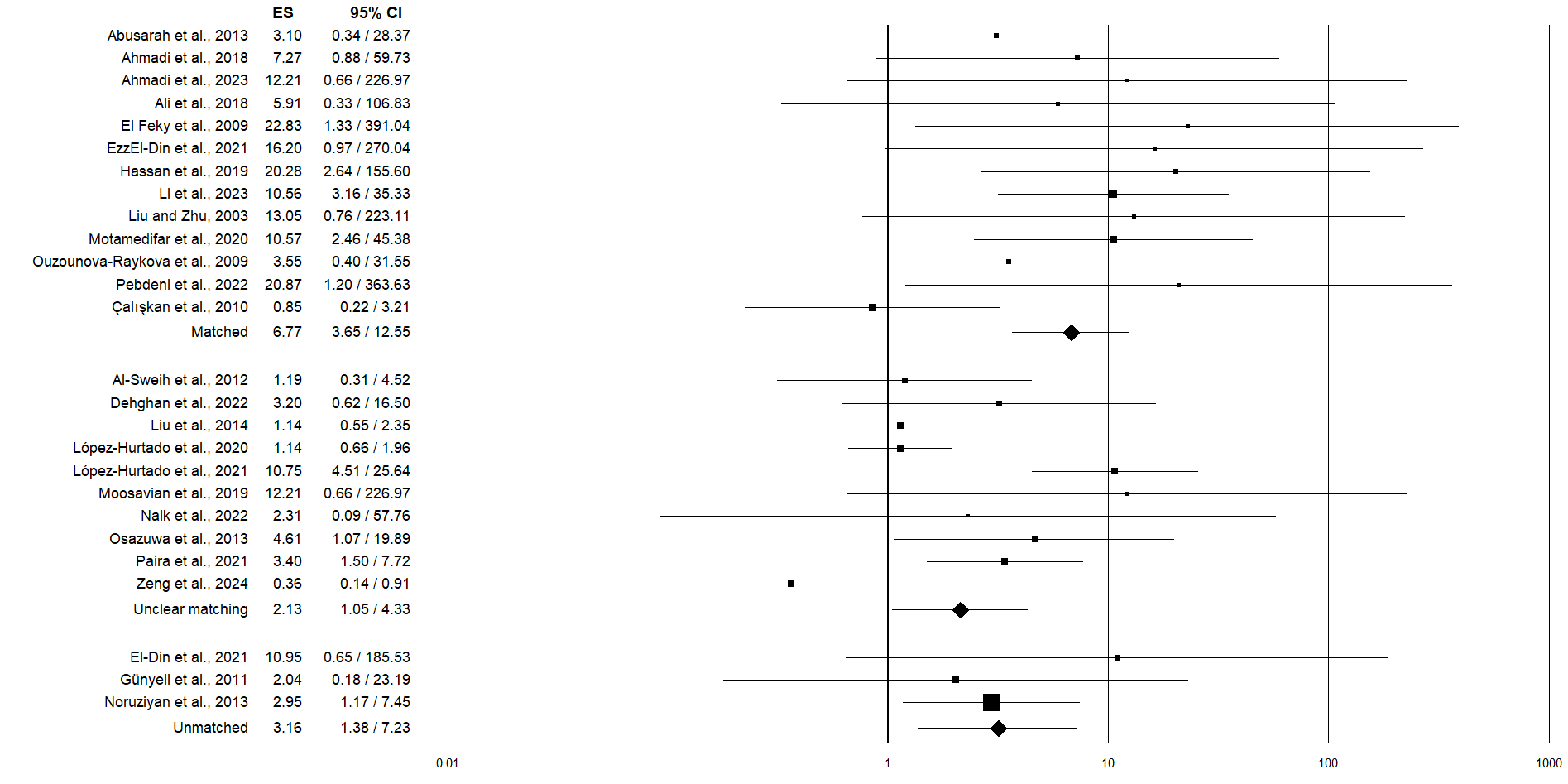


***This forest plot presents a subgroup analysis based on whether studies used age-matching for cases and controls. Studies are categorized into matched, unclear matching, and unmatched groups. Each study’s ES and corresponding 95%CI are displayed, with pooled estimates for each subgroup represented by diamonds. Larger squares indicate studies with greater weight in the analysis. The results show a marginally significant difference (p=0.047), with properly age-matched studies reporting a higher effect size (6.77 [95% CI 3.65–12.55]) compared to studies with unclear matching (2.13 [95% CI 1.05–4.33]) or no matching (3.16 [95% CI 1.38–7.23]). These findings suggest that age-matching may influence the observed association between Chlamydia trachomatis and male infertility.***

**Supplemental Figure 8. Subgroup analysis of effect sizes (ES) and 95% confidence intervals (CI) for the association between *Chlamydia trachomatis* and male infertility, stratified by a priori sample size calculation.**


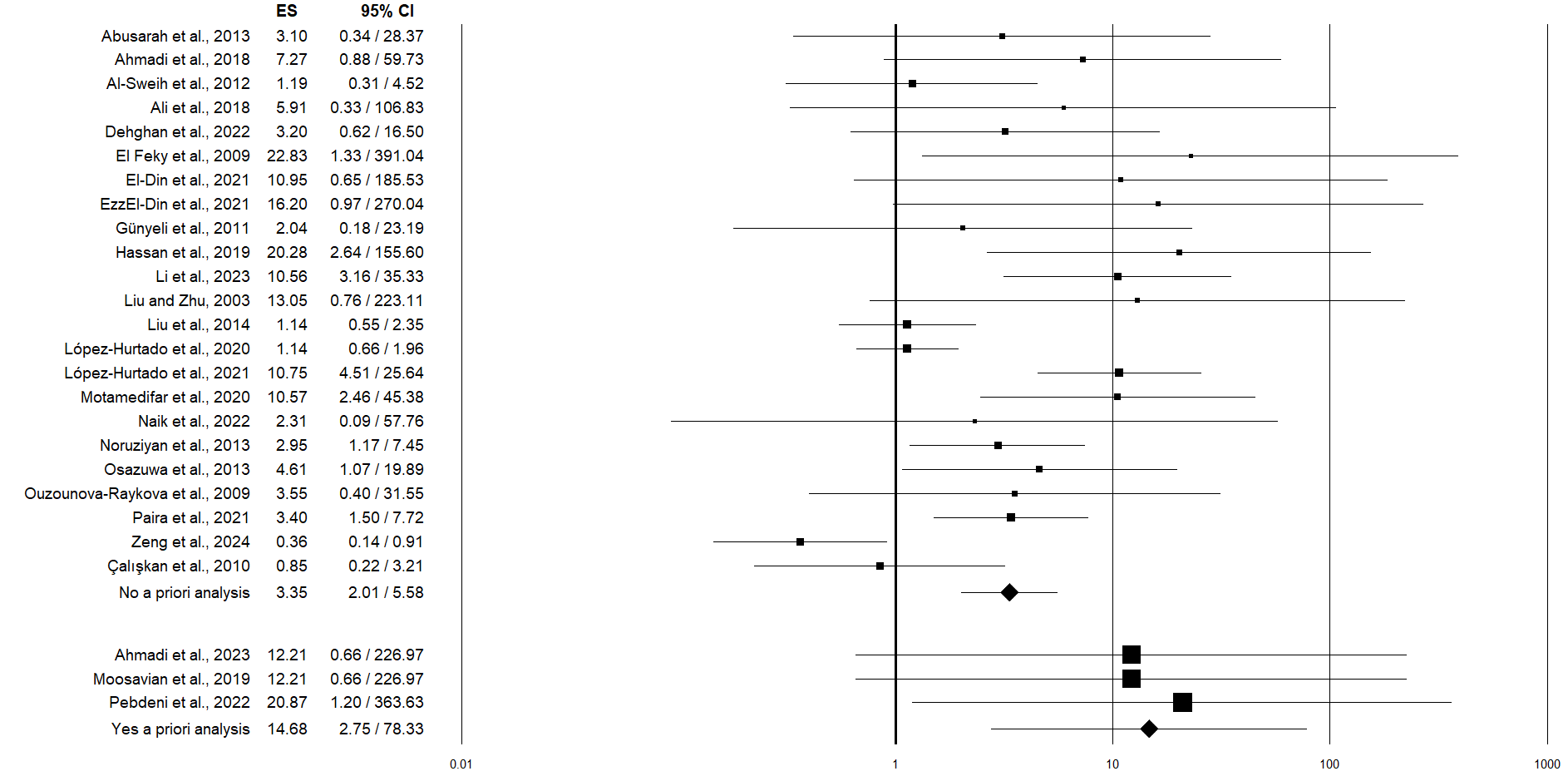


***This forest plot presents a subgroup analysis based on whether studies conducted an a priori sample size calculation. Studies are categorized into those that performed an a priori sample power analysis (“Yes”) and those that did not (“No”). Each study's ES and corresponding 95%CI are displayed, with pooled estimates for each subgroup represented by diamonds. Larger squares indicate studies with greater weight in the analysis. The results were found to be borderline significant (p=0.098), with studies that conducted an a priori sample size analysis reporting a higher effect size (14.68 [95% CI 2.75–78.33]) compared to those that did not (3.35 [95% CI 2.01–5.58]). However, the confidence interval for studies with a priori analysis is notably wide, suggesting higher variability in the effect estimate.***

**Supplemental Figure 9. Egger's regression test for publication bias.**


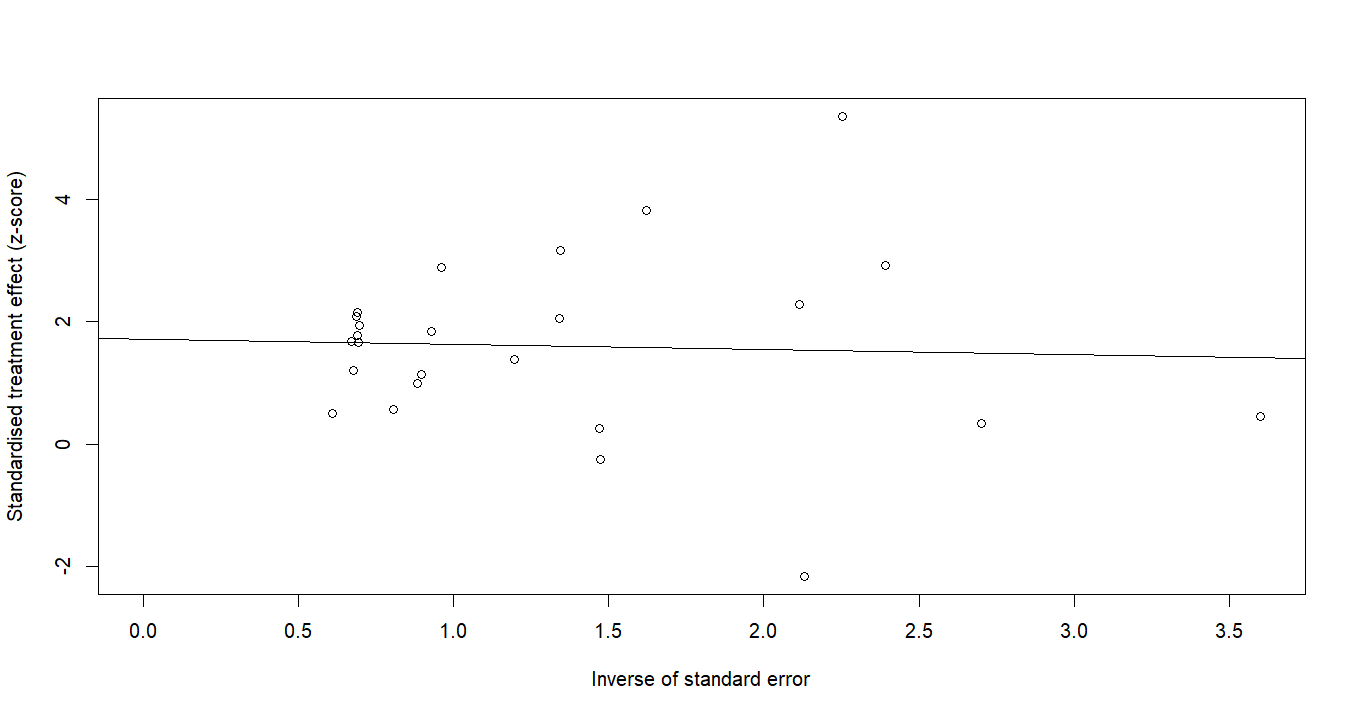


***This plot represents Egger's test, which assesses funnel plot asymmetry to detect potential publication bias in a meta-analysis. The x-axis shows the inverse of the standard error (precision), while the y-axis represents the standardized treatment effect (z-score). The regression line indicates the presence of asymmetry, suggesting bias (regression’s intercept=1.72, t=3.01, p=0.006).***
